# ST14 interacts with TMEFF1 and is a predictor of poor prognosis in ovarian cancer

**DOI:** 10.1186/s12885-024-11958-8

**Published:** 2024-03-11

**Authors:** Xin Nie, Lingling Gao, Mingjun Zheng, Shuang Wang, Caixia Wang, Xiao Li, Ouxuan Liu, Rui Gou, Juanjuan Liu, Bei Lin

**Affiliations:** 1 Department of Obstetrics and Gynecology, Shengjing Hospital of China Medical University, 36 Sanhao Road, Heping District, 110004 Shenyang, China; 2 Key Laboratory of Maternal-Fetal Medicine of Liaoning Province, Key Laboratory of Obstetrics and Gynecology of Higher Education of Liaoning Province, Shenyang, China; 3 Union Hospital, Tongji Medical College, Department of Obstetrics and Gynecology, Huazhong University of Science and Technology, Wuhan, China; 4 Department of Obstetrics and Gynecology, University Hospital, Ludwig-Maximilians-Universität München, Munich, Germany; 5 Department of Gynecology and Obstetrics, Tianjin Central Gynecology and Obstetrics Hospital Affiliated to Nankai University, Tianjin, China; 6 West China Second University Hospital, Department of Obstetrics and Gynecology, Sichuan University, Sichuan, China

**Keywords:** Prognostic indicator, Protein interactions, Ovarian cancer, ST14, TMEFF1

## Abstract

TMEFF1 is a new protein involved in the physiological functions of the central nervous system, and we previously reported TMEFF1 can promote ovarian cancer. ST14 was determined to be involved in the processes of epidermal differentiation, epithelial cell integrity, and vascular endothelial cell migration, etc. The relationship between ST14 and TMEFF1 in the ovary remains unknown. In this study, we detected the expression of ST14 and TMEFF1 in 130 different ovarian cancer tissues through immunohistochemistry. We determined ST14 and TMEFF1 were highly expressed in ovarian cancer, indicating a higher degree of tumor malignancy and a worse prognosis. Tissues significantly expressing ST14 also highly expressed TMEFF1, and the expression of the two proteins was positively correlated. Consistently, immunofluorescence double staining demonstrated the co-localization of ST14 and TMEFF1 in the same region, and immunoprecipitation confirmed the interaction between ST14 and TMEFF1. TMEFF1 expression was also reduced after knocking down ST14 through Western blot. MTT, wound healing and Transwell assays results determined that knockdown of ST14 inhibited proliferation, migration and invasion of ovarian cancer cells in vitro, but the inhibitory effect was restored after adding TMEFF1 exogenous protein. Gene Ontology and Kyoto Encyclopedia of Genes and Genomes pathways analysis showed that ST14 and its related genes were enriched in the processes of epithelial formation, intercellular adhesion, protein localization, and mitosis regulation. We also clarified the kinase, microRNA, and transcription factor target networks and the impact of genetic mutations on prognosis. Overall, high expression of ST14 and TMEFF1 in ovarian cancer predicts higher tumor malignancy and a worse prognosis. ST14 and TMEFF1 co-localize and interact with each other in ovarian cancer. ST14 can regulate TMEFF1 expression to promote proliferation, migration and invasion of ovarian cancer cells. We speculate that the relationship between ST14 and TMEFF1 in ovarian cancer could become a potential target for anti-cancer therapy.

**Supplementary Information:**

The online version contains supplementary material available at 10.1186/s12885-024-11958-8.

## Introduction

Ovarian cancer has the highest mortality rate among all gynecological tumors. Patients lack specific symptoms in the early stage, and up to 75% of patients are already in the late stage at the time of diagnosis. Thus, the 5-year survival rate is less than 50% [1, 2]. Therefore, finding effective biomarkers for screening and molecular targeted therapy is of great significance to improve the prognosis of this disease.

TMEFF1 (transmembrane protein with EGF-like and two follistatin-like domains) is a member of the Cancer testis antigens (CTAs) family, also known as tomoregulin-1 or TR-1 [4], encoded by the TMEFF1 gene located on chromosome 9q31 [3]. This protein contains a cytoplasmic C-terminal region, a transmembrane domain, two extracellular follistatin domains, and a modifiable EGF-like domain [4, 5]. Furthermore, it participates in physiological functions of the central nervous system, embryonic development, hair follicle regeneration and other biological processes [4–8]. In tumor research, TMEFF1 acts as a tumor suppressor gene in brain tumors [6]. High TMEFF1 expression has been detected in melanoma, liver cancer, and kidney cancer cell lines [9], but there have been no functional studies. In previous studies, we found that TMEFF1 is an oncogene in ovarian cancer [10].

ST14 (ST14 transmembrane serine protease matriptase), a member of the The type II transmembrane serine proteases (TTSPs) and also known as matriptase and MT-SP1 [11], is encoded by the ST14 gene located on chromosome 11q24-25. The ST14 protein consists of a shorter intracellular domain, a transmembrane domain, and a longer extracellular domain [12]. ST14 has been found to be involved in various physiological and pathological processes. It participates in epidermal differentiation [13, 14], the maintenance of epithelial cell integrity [15], and promoting vascular endothelial cell migration [16]. In tumors, ST14 promotes cell invasion, migration, and other malignant biological behaviors in breast cancer [17] and prostate cancer [18]. In autosomal recessive ichthyosis with hypotrichosis syndrome, ST14 was found to interact with TMEFF1 [19]. However, there has been no research on the function of ST14 and correlation between these two proteins in ovarian cancer. Therefore, in this study, we will explore the interaction between ST14 and TMEFF1 and their relationship with prognosis in ovarian cancer. The function of ST14-TMEFF1 in proliferation, invasion and metastasis of ovarian cancer will be detected by cytological experiments, which will provide a new research direction to explore the interaction between ST14 and TMEFF1 in ovarian cancer.

## Materials and methods

### Cell culture and gene transfection

Ovarian cancer cell lines SKOV3 and CAOV3 were purchased from the Institute of Biochemistry and Cell Biology, Chinese Academy of Sciences (Shanghai, China). Cells were routinely cultured in RPMI 1640 medium (GIBCO, USA, catalog number 10099-141) containing 10% fetal bovine serum at 37 °C with 5% CO_2_ and saturated humidity.

CAOV3 and SKOV3 cells in logarithmic growth phase were digested and seeded into 6-well plates. When cell confluency reached 50–70%, the siRNA fragments was transfected into the cells using Lipofectamine 3000 Transfection Kit (ThermoFisher). Two siRNAs showed synergic effects on the knockdown of ST14. The ST14 siRNA sequence 1 (Genepharma, China) was as follows: sense, 5′-GGGACUGGAUCAAAGAGAATT-3′; antisense, 5′- UUCUCUUUGAUCCAGUCCCTT-3′. The ST14 siRNA sequence 2 (Genepharma, China) was as follows: sense, 5′-GGAACAUUGAGGUGCCCAATT-3′; antisense, 5′-UUGGGCACCUCAAUGUUCCTT-3′.

### Specimen source and clinical data

The 130 ovarian tissue specimens included 91 cases of epithelial ovarian cancer (ovarian cancer group), 12 cases of ovarian epithelial borderline tumors (borderline group), 13 cases of ovarian epithelial benign tumors (benign group), and 14 cases of normal ovarian tissue (normal group). All ovarian tissues were obtained from paraffin blocks of the department of obstetrics and gynecology of our hospital from 2008 to 2016, and patients were re-diagnosed by pathologists. Patients in the malignant tumor group were 36–79 years of age, with a median age of 58 years; patients in the borderline tumor group were 30–66-years-old, with a median age of 46 years; patients in the benign tumor group were 30–68-years-old, with a median age of 42 years; and normal patients in the ovarian group were 35–64 years of age, with a median age of 45 years. There was no statistically significant difference among the ages of each group (*P* > 0.05). Nine cases of ovarian cancer were well differentiated, 35 were moderately differentiated, and 47 were poorly differentiated.

The stage was in accordance with the standards set by the FIGO in 2009 as follows: 35 cases were stage I-II, 56 were stage III-IV. Among them, 91 cases underwent comprehensive exploration and staging surgery in the early stage and cytoreductive surgery for ovarian tumors in the late stage. According to the pelvic and/or para-aortic lymph node metastasis, they were divided into 40 cases without metastasis, 28 cases with metastasis, and 23 cases without lymph dissection. None of the patients had received radiotherapy or chemotherapy before surgery [20].

### Immunohistochemistry

The sections of ovarian tissue in each group were 5 μm. The expression of ST14/TMEFF1 in ovarian cancer tissues was analyzed by immunohistochemical streptavidin-peroxidase staining (MXB Biotechnologies, China, catalog number KIT9720). The working concentrations of ST14 and TMEFF1 primary antibodies were 1:300 (Proteintech, rabbit, catalog number 27176-1-AP) and 1:200 (Santa Cruz, mouse, catalog number 393,457), respectively. Human pancreatic ductal adenocarcinoma tissue was used as a positive control for the ST14 antigen, and testicular tissue was used as a positive control for the TMEFF1 antigen. The negative control was incubated with IgG (ZSBIO, China, catalog number ZDR5006, ZDR5003) of the same species instead of the primary antibody. Yellow particles observed in the cytoplasm and cell membrane were considered a positive result. According to the coloring intensity, no staining, light yellow, brownish yellow, and tan were recorded as scores of 0, 1, 2, and 3, respectively. We selected five high-power fields from each section and then scored the percentage of stained cells, taking the average, as follows: less than 5% of chromatin cells = 0; 5–25% = 1; 26–50% = 2; 51–75% = 3; >75% = 4. These two numbers were multiplied, with the resulting classification as follows: 0–2 (-); 3–4, (+); 5–8, (++); and 9–12, (+++) as previously described [20–22]. Two pathologists independently scored samples to control for error.

### Double-labeling immunofluorescence method

The ovarian cancer cell lines CAOV3 were selected to make cell slides. ST14 and TMEFF1 double-labeling immunofluorescence was performed on cells and different ovarian tissue sections. The tissue sections and cells were incubated with primary antibodies against TMEFF1 (Santa Cruz, mouse, 1:50, catalog number 393,457) and anti-ST14 (Proteintech, rabbit, catalog number 27176-1-AP) at the same time as previously described [21, 22]. The primary antibody was replaced with rabbit or mouse IgG (Bioss, China, catalog number bs0296P, bs0295P) as a negative control (Figure S1). The working concentrations of fluorescein isothiocyanate and tetraethyl rhodamine isothiocyanate (ZSBIO, China, catalog number ZF0312, ZF0312) were 1:50. Samples were then incubated for 1 h at room temperature. The nucleus was counterstained with 4′,6-diamidino-2-phenylindole (DAPI) (Abcam, catalog number ab104139), and images were captured with a confocal microscope.

### Primary samples

Protein samples for western blotting were derived from tissue specimens collected at the Department of Obstetrics and Gynecology, Shengjing Hospital Affiliated to China Medical University, from 2021 to 2022. A total of 18 specimens were collected surgically, including 9 cases in the malignant group, and 9 cases in the normal group. All cases were newly diagnosed and have not received radiotherapy and chemotherapy. Every 3 samples of the same group were randomly mixed for western blot loading.

### Western blotting

Total protein extracted from ovarian cancer cells was quantified and denatured. The proteins were separated by 10% sodium dodecyl sulfate-polyacrylamide gel electrophoresis (SDS-PAGE) and transferred to a methanol-activated PVDF membrane (Millipore, catalog number IPVH00010). Antibody hybridization was performed after cutting the PVDF membrane to an appropriate size. After blocking with 5% milk for 1 h, the PVDF membrane was incubated with the primary antibody at 4 °C for 14 h. The primary antibodies were as follows: anti-TMEFF1 antibody (Santa Cruz, 1:500, catalog number 393,457), anti-ST14 antibody (Proteintech, rabbit, catalog number 27176-1-AP), anti-GAPDH (ZSBIO, China, 1:2000, catalog number TA08). After washing with TBST, the membrane was incubated with the secondary antibody (ZSBIO, China, 1:5000, ZB2301, ZB2305) at room temperature for 1.5 h. ECL luminescence reagent (Millipore, Billerica, MA, USA, catalog number WBKLS0500) was dropped onto the membrane, which was exposed for color development. The protein bands were visualized with Image J 1.31v software and normalized to the GAPDH protein expression level. Each experiment was repeated three times.

### Co-immunoprecipitation

Total protein from ovarian cancer cells was extracted, and 2 µg of anti-TMEFF1 monoclonal antibody (Santa Cruz, mouse, catalog number 393,457) or anti-ST14 polyclonal antibody (Proteintech, rabbit, catalog number 27176-1-AP) was added to the protein supernatant and incubated at 4 °C for 4 h. After adding 20 µL protein A/G PLUS-Agarose (Santa Cruz, catalog number sc2003), the sample was incubated overnight on a rocker platform at 4 °C as previously described [21, 22]. The primary antibody was replaced with IgG of the same species (Bioss, China, catalog number bs0296P, bs0295P) as a negative control. Subsequently, the immunoprecipitate was denatured and subjected to 10% SDS-PAGE gel electrophoresis. The subsequent experimental procedures were the same as those for western blotting. A TMEFF1 monoclonal antibody (Bioss, rabbit, catalog number bs17320R) or ST14 polyclonal antibody (Proteintech, mouse, catalog number 27176-1-ap) was used for incubation, and the experiment was repeated three times. Quantification of the micrographs fluorescence intensity was done via an ImageJ plug-in Colocalization Finder manager [23, 24].

### Transwell assay

Transwell chambers (Corning Costar, USA, catalog number 3421) were inoculated after being precoated via Matrigel (80ul). 2 × 105 cells in serum-free medium were transferred to the upper tier of the transwell chamber. 500ul 10% fetal bovine serum culture medium was added to the lower tier of the chamber and stayed at 37 °C for 48 h to facilitate cells to invade. The cells migrated to the lower surface were fixed with 4% paraformaldehyde and stained with 0.1% crystal violet. Stained cells in the entire field were counted under an inverted microscope.

### Wound healing assay

Cells were plated in 6-well plates at 1.25 × 105 cells/well overnight. A wound was scratched on the cell monolayer with a 200 µL sterile plastic tip. Cells were cultured in serum-free medium at 37 °C for 24 h, and then the wound healing processes were observed under a light microscope.

### MTT assay

CAOV3 cells and SKOV3 cells were seeded in a 96-well plate at 2000 cells/well. Cells adhering to the plate after 6 h were recorded as “0 h”. MTT solution (20 µl of 5 mg/mL, Solarbio, Beijing, China) was added to each well and incubated for 4 h. The medium was aspirated from each well, 150 µl DMSO was added followed by shaking for 10 min, and then the absorbance was measured (490 nm). The experiment was repeated at 24, 48, 72, 96 h. Set 5 repeat holes and set zero adjustment holes. The experiment was repeated three times [25].

### Oncomine database analysis

The Oncomine database (http://www.oncomine.org) [26, 27] has the most complete cancer mutation profile, gene expression data, and related clinical information, which can be used to discover new biomarkers or new therapeutic targets. The screening conditions in this study were as follows: ① “Cancer Type: Ovarian cancer;” ② “Gene: ST14;” ③ “Analysis Type: Cancer vs Normal Analysis;” ④ Critical value setting conditions (*P*-value < 0.05, fold-change > 2, gene rank = top 10%).

### UALCAN analysis

UALCAN (http://ualcan.path.uab.edu/analysis.html) [28] database is an effective online analysis and mining website for tumor data, mainly based on the clinical data of different cancer types in the TCGA database and TCGA 3 Level RNA-seq for analysis, biomarker identification, expression profile analysis, and subgroup analysis of related genes.

### GEPIA analysis

GEPIA (http://gepia.cancer-pku.cn/index.html) [29] integrates TCGA cancer data with GTEx normal tissue data, which can dynamically analyze gene expression profile data. We used the “General” module of this online analysis tool to analyze the expression level of the ST14 gene in ovarian cancer and other tumor tissues. The screening conditions in the “Expression DIY” module of this study were as follows: ①Gene: ST14; ② Datasets Selections: OV; ③ Log2FC Cutoff: 1; ④ *P*-value Cutoff: 0.01; analysis results. The expression original data used in the GEPIA website from UCSC Xena project (UCSC Toil RNA-seq Recompute, https://xenabrowser.net/datapages/), the involved original data in File S1.

### LinkedOmics analysis

The LinkedOmics database (http://www.linkedomics.org/login.php) [30, 31] is a web-based platform for analyzing 32 TCGA cancer-related dataset. The Pearson correlation coefficient was used to perform statistical analysis of ST14-co-expressed genes, which was displayed in the form of a volcano map, heat map, or scatter plot. The functional module of LinkedOmics uses gene set enrichment analysis (GSEA) to perform enrichment analysis on Gene Ontology (GO; cellular component, and molecular function), Kyoto Encyclopedia of Genes and Genomes (KEGG) pathway, kinase targets, miRNA targets, and transcription factor targets [32]. The grade standard was FDR < 0.05, and 500 simulations were carried out.

### Metascape

Metascape (http://metascape.org) [33] is a free, user-friendly gene list analysis tool for gene annotation and analysis. In this study, Metascape was used for pathway and process enrichment analysis of ST14 and its significantly related genes. The GO terms for biological process, cellular component, and molecular function categories, as well as KEGG pathways, were enriched based on the Metascape online tool. Only a *P*-value < 0.01, a minimum count of 3, and an enrichment factor > 1.5 were considered statistically significant [20].

### cBioPortal analysis

cBioPortal (www.cbioportal.org) [34, 35] is an online open website for analyzing and visualizing multidimensional cancer genomics data. We selected data from ovarian cancers in the “Query” section, and entered ST14 in “Query by Gene” section, used cBioPortal for further analysis. The type and frequency of ST14 gene mutation in ovarian cancer were analyzed in “OncoPrint”. “OncoPrint” shows the mutation, copy number, and expression of the target gene in all samples in the form of a heat map. In this study, we analyzed the ST14 gene mutation. A Kaplan–Meier diagram was used to show the association between ST14 gene mutations and overall survival (OS), disease-free survival (DFS), disease-specific survival (DSS), and progression-free survival (PFS) in ovarian cancer patients, and the log-rank test was performed. *P* < 0.05 was considered a significant difference [20].

### GeneMANIA analysis

GeneMANIA (http://www.genemania.org) [36] is an online platform that analyzes and displays genes that perform similar functions—showing the interaction between protein expression and genetics in the network.

### STRING analysis

The STRING database (https://string-db.org) [37] is a database containing vast amounts of protein-protein interaction (PPI) data. We used it to construct the PPI network of ST14.

### Statistical analysis

Using the SPSS22.0 software system, counting data were subjected to a x^2^ test and Fisher’s exact probability test, whereas measurement data were subjected to one-way analysis and Student’s *t* test of variance. The Cox regression model was used to analyze risk factors. Kaplan–Meier and log-rank methods were used to analyze and compare survival curves. Spearman correlation analysis and the regression model were used to analyze correlations between the two proteins. *P* < 0.05 was regarded as statistically significant.

## Results

### Analysis of ST14 expression in Oncomine, UALCAN, and GEPIA databases and ovary tissues

**Fig. 1:**
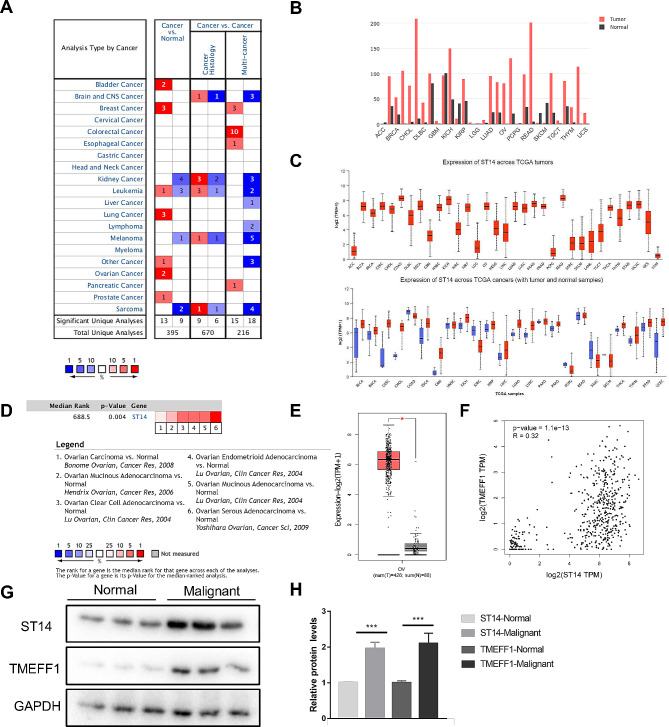
ST14 expression in different datasets from patients with ovarian cancer. (**A**) Oncomine analysis of the mRNA expression levels of ST14 genes in different cancers. The differences in expression levels of genes between cancer and normal tissues are concluded. The thresholds are indicated in the colored cells. *P* < 0.05, fold-change > 2 and gene rank = 10% were considered statistically significant. Red cells represent overexpression of the target gene in tumor tissues compared to normal tissues, while blue cells indicate downregulation of the gene. Gene rank is depicted by the color depth in the cells. (**B**) UALCAN analysis of the mRNA expression levels of ST14 genes in different cancers. (**C**) GEPIA analysis of the mRNA expression levels of ST14 genes in different cancers. (**D**) ST14 DNA copy numbers based on chips for ovarian cancer research in TCGA Ovary. *P < 0.05. (**E**) Levels of ST14 mRNA in ovarian cancer based on research in the GEPIA websites (red for tumor, black for normal). The boxplot analysis showed the expression level by log2 (TPM + 1) on a log-scale. *P < 0.05. (**F**) Correlation between ST14 and TMEFF1 expression in ovarian cancer based on the GEPIA website. *R* = 0.32, ***P < 0.001. (**G**) The expression of ST14 and TMEFF1 in ovarian malignant tumor tissues and normal tissues detected by western blot. (**H**) Quantification of TMEFF1 normalized to GAPDH. Data are presented as the mean ± SEM (*n* = 3 per group). *P < 0.05, **P < 0.01, and ***P < 0.001

The research results regarding ST14 expression in 395 different types of tumors have been collected in the Oncomine database. There are 21 research results showing statistical differences in ST14 mRNA levels. Among them, there are 13 in which the expression of ST14 mRNA was significantly increased, and nine in which the expression was significantly reduced. The expression of ST14 mRNA was significantly increased in bladder cancer, breast cancer, lung cancer, ovarian cancer, prostate cancer, and other cancer, and the expression was decreased in kidney cancer, melanoma, and sarcoma (Fig. 1A). UALCAN and GEPIA website analysis showed that the expression of ST14 mRNA was significantly increased in breast invasive carcinoma, cholangiocarcinoma, lymphoid neoplasm diffuse large B-cell lymphoma, lung adenocarcinoma, ovarian serous cystadenocarcinoma, testicular germ cell tumor, thymoma, and uterine carcinosarcoma, among others and decreased in skin cutaneous melanoma (Fig. 1B, C). To further study the expression of ST14 based on different ovarian cancer research chips, we used the Oncomine database to identify six datasets containing ST14 expression data. All showed that compared to levels in normal tissues, ST14 is overexpressed in ovarian cancer (*P* = 0.004; Fig. 1D). The GEPIA website was further used to analyze the expression of ST14 in 426 ovarian cancer specimens and 88 normal ovarian specimens, and results suggested that ST14 mRNA was significantly highly expressed in ovarian cancer (*P* < 0.05; Fig. 1E). Through analysis of the GEPIA website, it was also found that in ovarian cancer (TCGA tumor) and normal ovaries (GTEx), the expression levels of TMEFF1 and ST14 are positively correlated (*R* = 0.32, *P* = 1.1e-13; Fig. 1F). We collected ovarian tissue from 18 clinical patients (9 cases of ovarian cancer and 9 cases of normal ovary tissue) to verify the expression of ST14 and TMEFF1 by western blot, and we found that the expression of ST14 and TMEFF1 in ovarian cancer was significantly higher than that in normal tissue (P both < 0.001; Fig. 1G-H).

### Analysis of ST14 expression with UALCAN

**Fig. 2:**
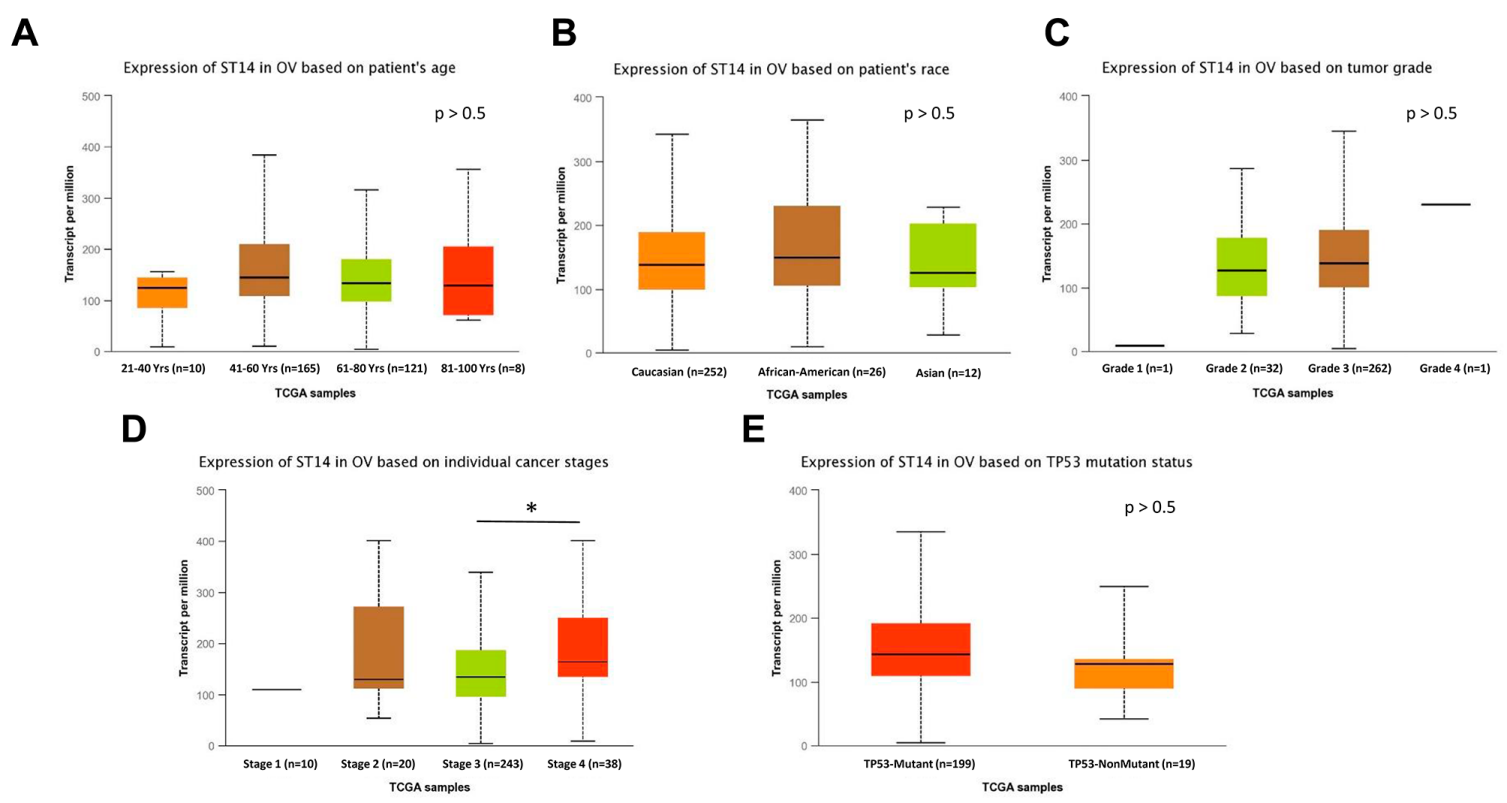
Levels of ST14 in subgroups of patients with ovarian cancer. Levels of ST14 expression in ovarian cancer patients based on different (**A**) ages, (**B**) races, (**C**) tumor grades, (**D**) cancer stages, and (**E**) TP53 methylation statuses. OV, ovarian serous cystadenocarcinoma. *P < 0.05

Further, through the UALCAN online analysis website, subgroup analysis of 301 cases of ovarian serous cystadenocarcinoma was performed based on various clinicopathological characteristics in TCGA. The expression of ST14 was not significantly different based on age (Fig. 2A), patient race (Fig. 2B), and grade (Fig. 2C). However, based on different cancer stages, the expression of ST14 increased with a higher stage, and the expression in stage 4 was determined to be significantly higher than that in stage 3 (*P* < 0.05; Fig. 2D). The expression of ST14 in TP53-mutation-positive disease was higher than that in the TP53-non-mutation group, but was not statistically different due to the small number of cases (Fig. 2E).

### Enrichment analysis of ST14 functional networks in ovarian cancer

**Fig. 3:**
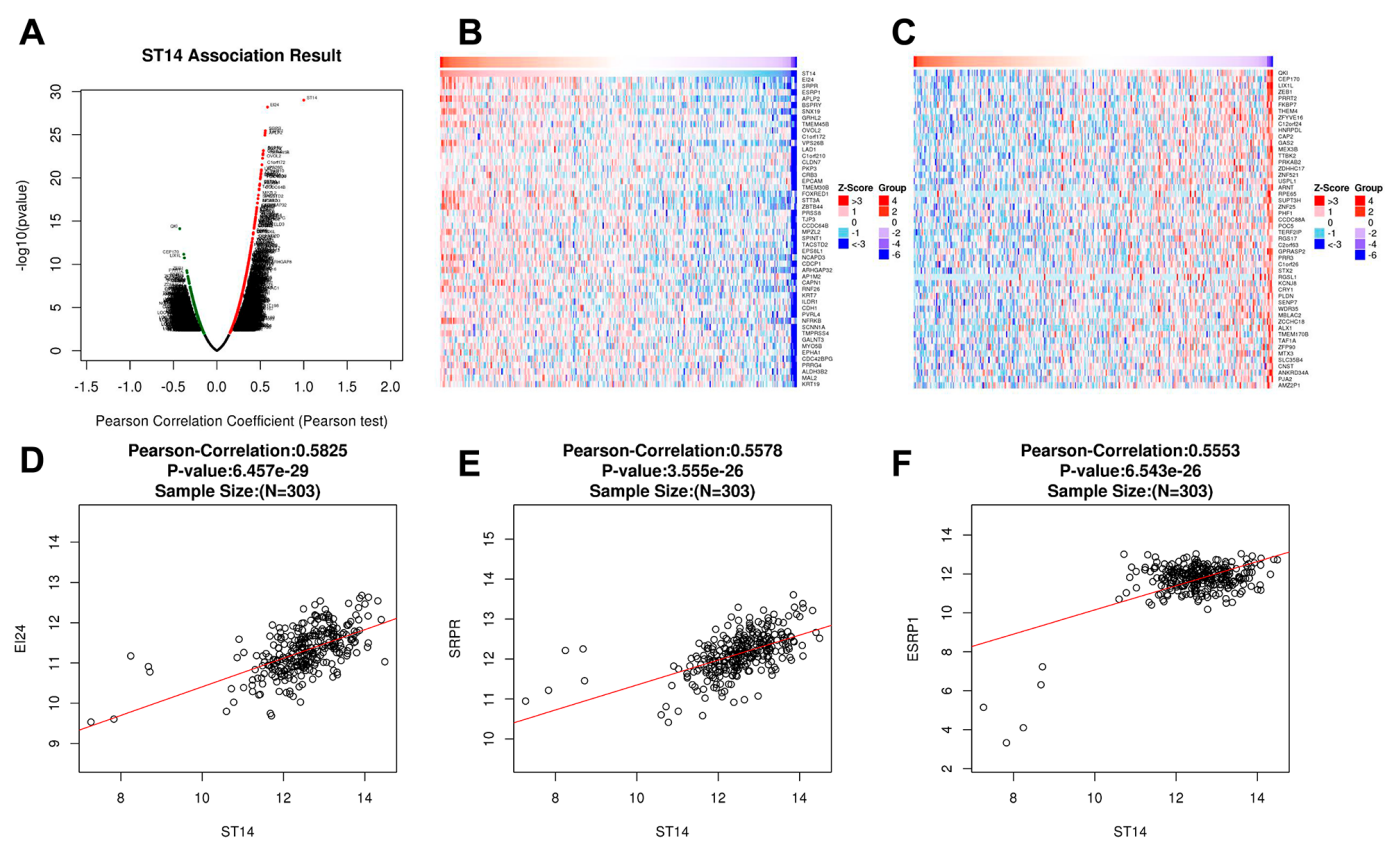
Differentially expressed genes correlated with ST14 in ovarian cancer. (**A**) Correlations between ST14 and genes differentially expressed in ovarian cancer were assessed by the Pearson test. (**B**) Genes positively correlated with ST14 in ovarian cancer as heat maps (TOP 50). Red: positively correlated genes. Blue: negatively correlated genes. (**C**) Genes negatively correlated with ST14 in ovarian cancer as heat maps (TOP 50). (**D**-**F**) Correlation between ST14 expression and the expression of EI24 (**D**), SRPR (**E**), and ESRP1 (**F**) based on the Pearson test, shown with a scatter plot (*P* = 6.457e-29, *P* = 3.555e-25, *P* = 6.453e-26, respectively)

We next used the function module in LinkedOmics to analyze the mRNA sequencing data of 303 ovarian cancer patients in the TCGA database. As shown in the volcano map, there were 813 genes that were significantly positively related to ST14 (dark red dots) and 568 genes that were significantly negatively related to ST14 (dark green dots) (false discovery rate [FDR] < 0.01; Fig. 3A). The heat map shows the first 50 gene sets that were significantly positively (Fig. 3B) and negatively (Fig. 3C) correlated with ST14. The result indicated that ST14 has a wide range of effects on epithelial cell formation, cell–cell connections, and cell migration, among others. The statistical scatter plot of a single gene showed that the expression of ST14 was significantly positively correlated with EI24, SRPR, and ESRP1 (*P* < 0.001, Fig. 3D-F). These genes play an important role in inhibiting growth, regulating the process of autophagy, regulating nascent secretory proteins targeting the endoplasmic reticulum system, and regulating the formation of epithelial cell-specific isoforms.

### GO and KEGG enrichment analysis of ST14 functions and related differentially expressed genes

**Fig. 4:**
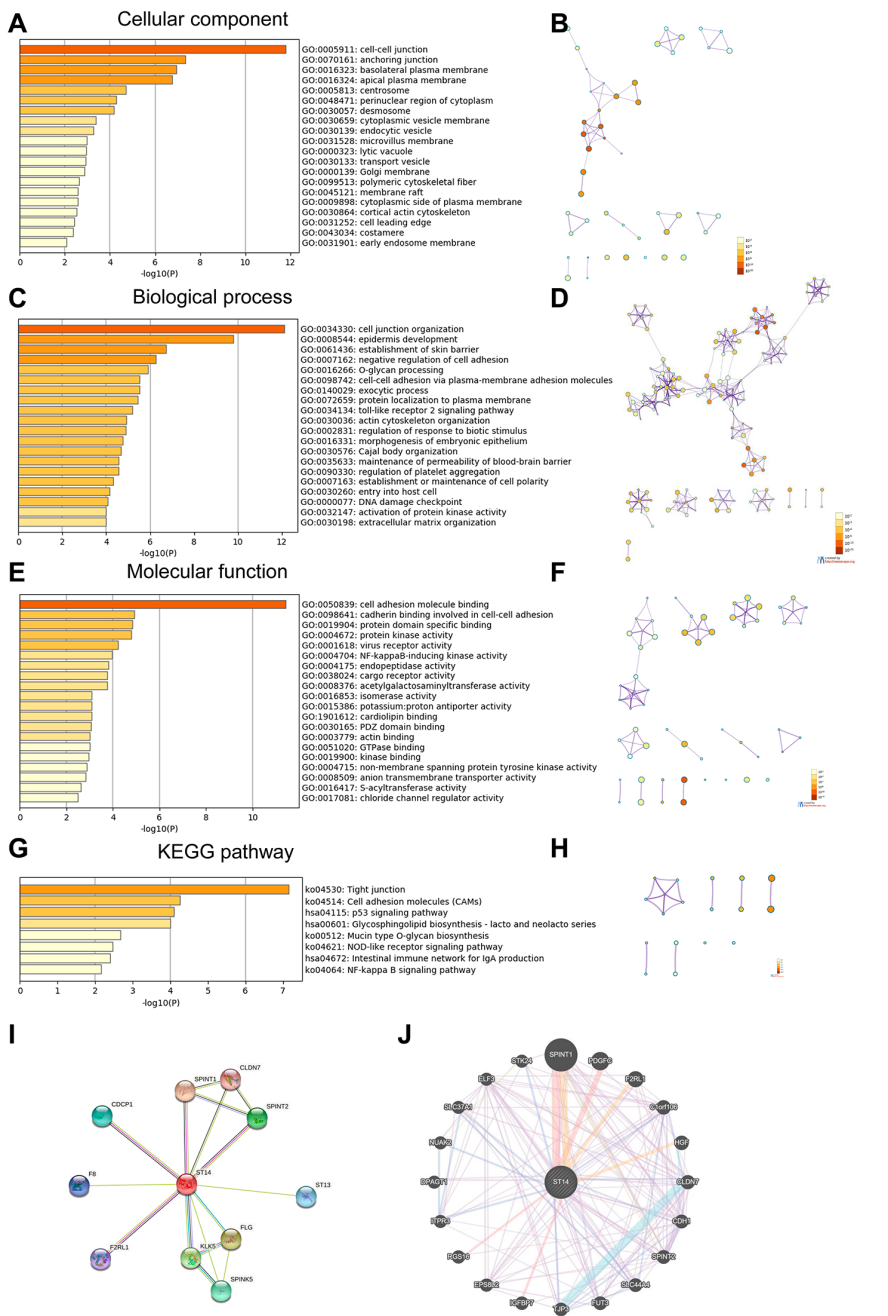
Significantly enriched GO annotations and KEGG pathways of ST14-co-expressed genes and proteins interacting with ST14 in ovarian cancer. Results were analyzed with Metascape. The top 20 enriched (**A**) cellular components, (**C**) biological processes, and (**E**) molecular functions related to ST14-related genes are shown, with the bar graph colored based on *P*-values. (**B**, **D**, **F**) Network of GO-enriched terms colored based on the *P*-value, where terms containing more genes tended to have a more significant *P*-value. (**G**) KEGG-enriched terms colored based on *P*-values. (**H**) Network of KEGG-enriched terms colored based on the *P*-value, where terms containing more genes tended to have a more significant *P*-value. GO, Gene Ontology; KEGG, Kyoto Encyclopedia of Genes and Genomes. (**I**) PPI analysis using the STRING database. (**J**) PPI analysis using the GeneMANIA database

GO results showed that ST14 and its related differentially expressed genes are mainly located in the cell–cell junction, anchoring junction, basolateral plasma membrane, apical plasma membrane, centrosome, and other structures (Fig. 4A, B and Additional file 1: Table S1). Further, they were mainly involved in the formation of the epithelium, cell adhesion, protein localization, mitosis regulation, and other biological processes, such as cell junction organization, epidermis development, establishment of skin barrier, negative regulation of cell adhesion, O-glycan processing, cell–cell adhesion via plasma membrane adhesion molecules, exocytic process, protein localization to the plasma membrane, and DNA damage checkpoint, among others (Fig. 4C, D and Additional file 1: Table S2). The molecular functions of ST14 and related genes mainly included regulating the activities of protein kinases, virus receptor, NF-kappa B-inducing kinase, cargo receptor endopeptidase, isomerase, and acetylgalactosaminyltransferase, among others, and can be combined with cell adhesion molecules, cadherin, cardiolipin, PDZ domains, and actin, among others (Fig. 4E, F and Additional file 1: Table S3).

KEGG enrichment analysis results showed that ST14 and its related differentially expressed genes participate in signaling pathways, including tight junction, cell adhesion molecules (CAMs), p53, glycosphingolipid biosynthesis-lacto and neolacto series, mucin type O-glycan biosynthesis, NOD-like receptor, intestinal immune network for IgA production, and NF-kappa B, among others (Fig. 4G, H and Additional file 1: Table S4). And the aforementioned signaling pathways can participate in the occurrence and development of a variety of tumors and are closely related to the occurrence and development of ovarian cancer.

### ST14 network of kinases, miRNA, or transcription factor targets in ovarian cancer

To further explore the targets of ST14 in ovarian cancer, we analyzed the kinase, miRNA, and transcription factor target networks of the positively related gene set generated by GSEA. The top five most important kinase target networks were Kinase_EGFR, Kinase_DYRK1A, Kinase_PRKCA, Kinase_SRC, and Kinase_MAP2K6 (Table 1 and Additional file 1: Table S5-S7), which were mainly related to cell growth, regulation of nuclear functions of cell proliferation, regulation of synaptic plasticity, control of the transduction of various biological signals (cell adhesion, cell cycle progression, apoptosis, migration and transformation, etc.), regulation of cell response to cytokines, and various excited reactions, among others. The miRNA target network included TTTGTAG, MIR-520D, AGTCTTA, MIR-499, CATGTAA, MIR-496, GTATTAT, MIR-369-3P, GTACTGT, and MIR-101. The transcription factor target network included V$PEA3_Q6, KMCATNNWGGA_UNKNOWN, V$AREB6_01, V$AP1_Q4_01, and V$TEF1_Q6, which are mainly related to transcription activation, transcription repression, regulation of protein sorting in the late-Golgi/trans-Golgi network and endosomes, and regulation of tumor-related Hippo signaling pathways, among others.

**Table 1:** The kinase, miRNA, and transcription factor-target networks of ST14 in ovarian cancer

Enriched category	Gene set	Leading edge number	FDR
Kinase target	Epidermal growth factor receptor	12	0.18957
	Dual specificity tyrosine Phosphorylation regulated kinase 1 A	7	0.24991
	Protein kinase C alpha	62	0.37146
	SRC proto-oncogene, non-receptor tyrosine kinase	49	0.41552
	Mitogen-activated protein kinase kinase 6	2	0.41843
miRNA target	TTTGTAG, MIR-520D	109	0
	AGTCTTA, MIR-499	18	0
	CATGTAA, MIR-496	68	0
	GTATTAT, MIR-369-3P	77	0
	GTACTGT, MIR-101	64	0.0033256
Transcription factor target	V$PEA3_Q6	90	0
	KMCATNNWGGA_UNKNOWN	36	0
	V$AREB6_01	46	0.0027337
	V$AP1_Q4_01	62	0.0061963
	V$TEF1_Q6	40	0.0069253

### PPI analysis using the STRING database and GeneMANIA database

To better understand the role of ST14 in ovarian cancer, we analyzed the genes most relevant with ST14 and construct the PPI network using GeneMANIA and STRING database (Fig. 4I, J). The results show that proteins that interact with ST14 are involved in cell-cell junction organization and maintenance, regulation of phosphatidylinositol 3-kinase signaling, terminal differentiation of epidermis, inhibition of various enzymatic activities, integrity and protective barrier function of the skin, regulation of blood coagulation, and various signal transduction processes.

### Genome variations in ST14 in ovarian cancer

**Fig. 5:**
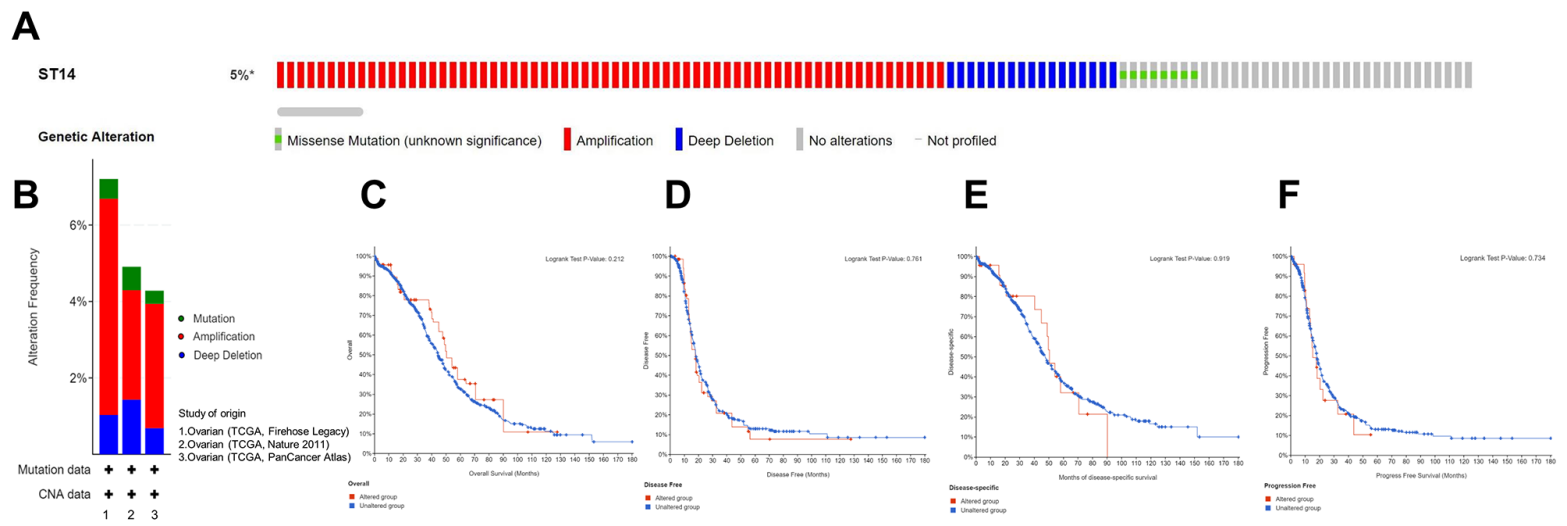
Analysis of ST14 genetic variations and effect on survival and prognosis of ovarian cancer patients. (**A**) Mutations in the ST14 gene based on the cBioPortal database. (**B**) Analyses of genetic variations in ST14 reported in different studies. The variations included mutation (green), amplification (red), and deep deletions (blue). TCGA: The Cancer Genome Atlas. (**C**-**F**) Effect of mutations in the ST14 gene on the (**C**) overall survival (OS), (**D**) disease-free survival (DFS), (**E**) disease-specific survival (DSS), and (**F**) progression-free survival (PFS) of ovarian cancer patients (*P* > 0.05)

We used cBioPorta to analyze the genetic variations in ST14 in 1680 ovarian serous cystadenocarcinoma patients retrieved from three studies (TCGA, Firehose Legacy; TCGA, Nature 2011; TCGA, PanCancer Atlas) (File S2). ST14 gene mutations were present at a low incidence in ovarian serous cystadenocarcinoma. Among the 1680 ovarian serous cystadenocarcinoma patients, only 89 (5.3%) had mutations in the ST14 gene (Fig. 5A, B), and the type and frequency were as follows: amplification, 64 cases (3.8%); deep mutation, 17 cases (1.0%); missense mutation (unknown significance), eight cases (0.5%). In addition, ST14 gene mutations had no significant effect on OS, DFS, PFS, and DSS for patients with ovarian serous cystadenocarcinoma (Fig. 5C-F).

### Expression patterns of ST14 and TMEFF1 in clinical patient ovarian tissue groups

**Fig. 6:**
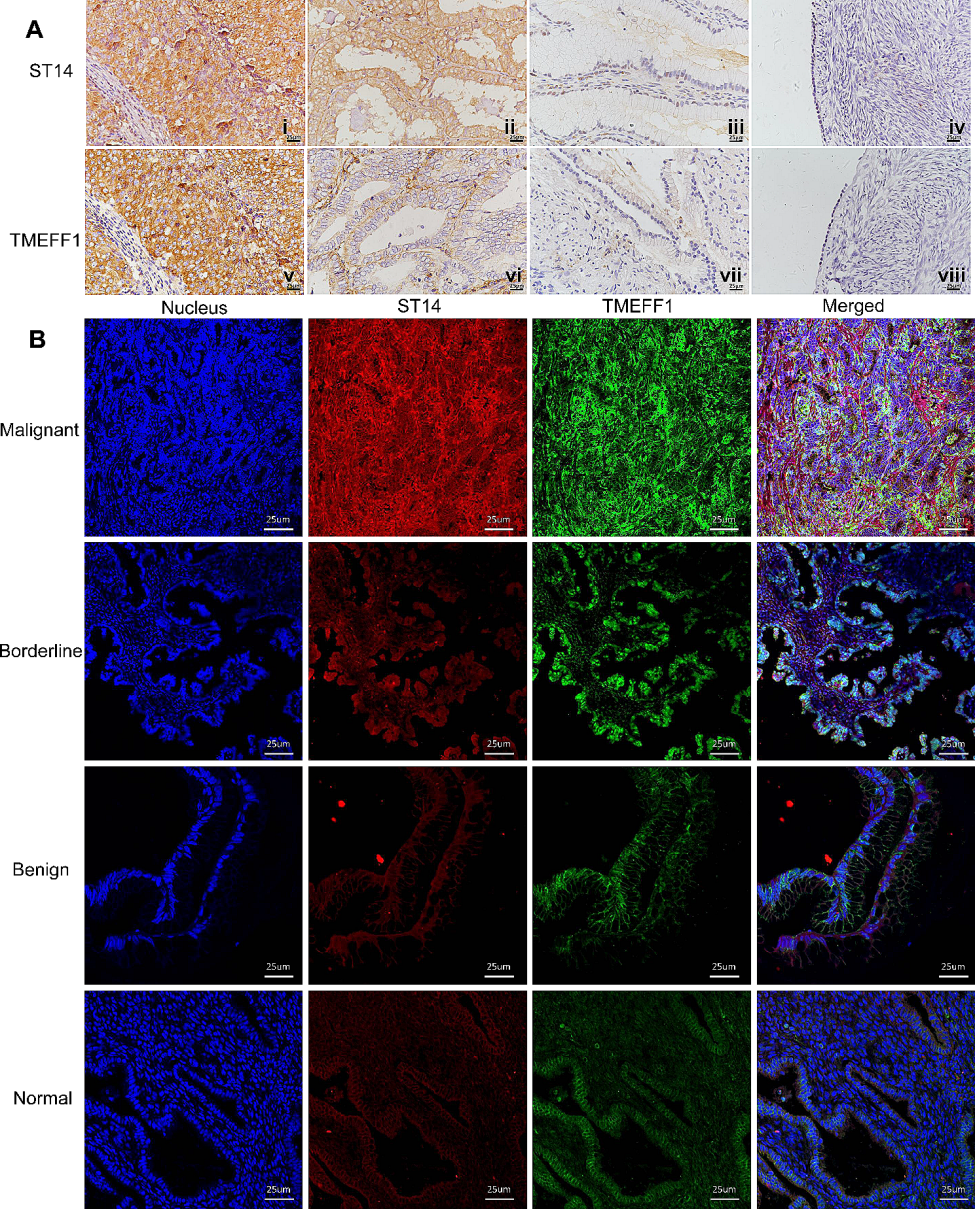
Expression and co-localization of ST14 and TMEFF1 in different ovarian tissues. (**A**) Immunohistochemical staining of ovarian malignant tumors (i, v), borderline tumors (ii, vi), benign tumors (iii, vii), and normal ovarian tissues (vi, viii). ST14 (i-iv) and TMEFF1 (v-viii) staining is shown (original magnification, ×400). (**B**) Dual-labeled immunofluorescence technology was used to detect the co-localization of ST14 and TMEFF1 in different ovarian tissues. Blue represents the nucleus, red represents ST14, green represents TMEFF1, orange represents the co-localization of ST14 and TMEFF1 (original magnification, ×400). Pearson’s correlation coefficient (Rr) and Manders’ overlap coefficient (R) of the co-localization images: malignant tumors (Rr:0.64, R:0.87), borderline tumors (Rr:0.76,R:0.79), benign tumors (Rr:0.63,R:0.68), and normal ovarian tissues (Rr:0.76,R:0.92)

The staining of ST14 and TMEFF1 mainly occurred in the cell membrane and cytoplasm (Fig. 6A).

The positive expression and high expression rates of ST14 in ovarian cancer were 93.4% (85/91) and 73.6% (67/91), respectively, which were significantly higher than those in normal ovarian epithelial tissue, specifically 21.4% (3/14) and 14.3% (2/14) (*P* < 0.001, 0.001), respectively; moreover, the high expression rate was higher than that in benign tumors, (38.5%; 5/13; *P* = 0.01). In addition, the ST14 positive expression rates in borderline ovarian tumors and benign tumors were 83.3% (10/12) and 76.9% (10/13), which were significantly higher than those in normal ovarian epithelial tissues (*P* = 0.002, 0.007; Fig. 6A; Table 2). The positive and high expression rates of TMEFF1 in ovarian cancer were 89.0% (81/91) and 63.7% (58/91), respectively, which were significantly higher than those in borderline tumors, 58.3% (7/12) and 33.3% (4/12) (*P* = 0.014, 0.06), benign tumors, 38.5% (5/13) and 15.4% (2/13) (*P* < 0.001, 0.002), and normal ovarian epithelial tissue, 28.6% (4/14), and 7.1% (1/14) (*P* < 0.001, 0.001; Fig. 6A; Table 3).

**Table 2:** Expression of ST14 in different ovarian tissues

Groups	Cases	(-)	(+)	(++)	(+++)	Positive rate%	High expression rate%
Normal	14	11	1	2	0	21.4	14.3
Benign	13	3	5	4	1	76.9**	38.5
Borderline	12	2	4	5	1	83.3**	50.0
Malignant	91	6	18	29	38	93.4***	73.6***

Note: **P < 0.01, ***P < 0.001

**Table 3:** Expression of TMEFF1 in different ovarian tissues

Groups	Cases	(-)	(+)	(++)	(+++)	Positive rate%	High expression rate%
Normal	14	10	3	1	0	28.6	7.1
Benign	13	8	3	1	1	38.5	15.4
Borderline	12	5	3	3	1	58.3	33.3
Malignant	91	10	23	34	24	89.0***	63.7***

Note: ***P < 0.001

### Relationship between the expression of ST14/TMEFF1 and clinicopathologic parameters of ovarian cancer

This study included 91 cases of ovarian cancer. The high expression rate of ST14 in early stage (I-II) was 54.3% (19/35), which was significantly lower than that in advanced stage (III-IV), specifically 85.7% (48/56) (*P* < 0.001). Similar to ST14, the high expression rate of TMEFF1 in early stage (I-II) was 47.7% (15/35), which was significantly lower than that in advanced stage (III-IV), 77.0% (43/56) (*P* < 0.001). The ST14 positive expression rate in the poorly differentiated group was 83.0% (39/47), which was significantly higher than that in the high–medium differentiated group (63.6%; 28/44; *P* = 0.036). The positive expression rate of TMEFF1 in the lymph node metastasis group was 85.7% (24/28), which was significantly higher than that in the non-lymph node metastasis group (45.0%; 18/40; *P* < 0.001). The expression of ST14 showed no obvious relationship with lymph node metastasis and clinicopathologic characteristics of the tumors; the expression of TMEFF1 also had no obvious relationship with differentiation and clinicopathologic characteristics of the tumors (Tables 4 and 5).

**Table 4:** Association between ST14 expression and pathological features in ovarian cancer

Features	Cases	High expression cases	High expression rate%	*P*-value
**FIGO stage**				0.001***
I-II	35	19	54.3	
III-IV	56	48	85.7	
**Differentiation**				0.036*
Well-moderate	44	28	63.6	
Poorly	47	39	83.0	
**LN** **metastasis**				*0.394*
No	40	28	70.0	
Yes	28	23	82.1	
no lymphadenectomy	23	16	69.6	
**Pathologic type**				*>0.05*
Serous	38	28	73.7	
Mucinous	11	7	63.6	
Endometrioid	18	12	66.7	
Clear cell carcinoma	8	6	75.0	
Poorly differentiated adenocarcinoma	16	14	87.5	

**Notes**: *P < 0.05, ***P < 0.01

**Abbreviations**: FIGO, International Federation of Gynecology and Obstetrics; LN,lymph node

**Table 5:** Association between TMEFF1 expression and pathological features in ovarian cancer

Features	Cases	High expression cases	High expression rate%	*P*-value
**FIGO stage**				0.001***
I-II	35	15	47.7	
III-IV	56	43	77.0	
**Differentiation**				*0.575*
Well-moderate	44	30	68.1	
Poorly	47	28	59.6	
**LN** **metastasis**				0.001***
No	40	18	45.0	
Yes	28	24	85.7	
no lymphadenectomy	23	16	69.6	
**Pathologic type**				*>0.05*
Serous	38	25	65.8	
Mucinous	11	7	63.6	
Endometrioid	18	10	55.5	
Clear cell carcinoma	8	5	62.5	
Poorly differentiated adenocarcinoma	16	11	68.8	
**Notes**: ***P < 0.01				

**Abbreviations**: FIGO, International Federation of Gynecology and Obstetrics; LN, lymph node

### ST14 and TMEFF1 overexpression in ovarian cancer predicts patient survival

A follow-up of patients with ovarian cancer (as of January 30, 2020) with univariate Kaplan–Meier analysis showed that the high expressions of ST14 and TMEFF1 were both correlated with shortened OS (*P* = 0.003; 0.001, respectively). The average survival time for the low expression group was 56.7 months, whereas the average survival time for the ST14-high expression group was 45.9 months. The average survival time of the low expression group was 58.5 months, whereas the average survival time of the TMEFF1-high expression group was 46.0 months. In addition, FIGO stage (I ~ II versus III ~ IV) was also associated with poor prognosis (*P* = 0.008; Table 6). Cox regression model was used to analyze the relationship between the prognosis of ovarian cancer patients and different clinicopathological parameters, it was found that FIGO stage, ST14 expression, and TMEFF1 expression affected the survival time (*P* = 0.012, 0.005, 0.001, respectively). Multivariate Cox regression analysis found that ST14 expression and TMEFF1 expression were independent risk factors affecting the prognosis of patients with ovarian cancer (*P* = 0.026, 0.002; Table 7). In summary, this shows that ST14 and TMEFF1 can effectively predict the prognosis of ovarian cancer patients.

**Table 6:** Univariate Kaplan-Meier prognostic analysis of ovarian cancer

Variable	Characteristics	(Log-rank) *P*-value
Age at diagnosis	<50 years vs. ≥ 50 years	*0.651*
FIGO stage	I-II vs. III-IV	0.008**
Differentiation grade	Well-moderate vs. poor	*0.508*
LN metastasis	Negative vs. positive	*0.146*
ST14	Low vs. high	0.003**
TMEFF1	Low vs. high	0.0001***

**Notes**: **P < 0.01, ***P < 0.001

**Table 7:** Univariate and multivariate Cox regression analysis of patients with ovarian cancer

Variables	Univariate analysis			Multivariate analysis	
	*P*-value	Hazard ratio (95% CI)		*P*-value	Hazard ratio (95% CI)
Age at diagnosis	*0.658*	1.171 (0.582–2.355)		*0.168*	0.594 (0.283–1.2477)
FIGO stage	0.012*	2.699 (1.239–5.877)		*0.103*	1.957 (0.874–4.383)
Differentiation grade	*0.526*	1.769 (0.614–5.092)		*0.993*	1.002 (0.621–1.618)
LN metastasis	*0.171*	1.864 (0.784–4.430)		*0.277*	1.270 (0.825–1.953)
ST14	0.005**	2.862 (1.381–5.931)		0.026*	2.317 (1.105–4.859)
TMEFF1	0.001***	6.578 (2.323–18.626)		0.002**	5.531 (1.863–16.419)

Note: **P < 0.01, ***P < 0.001

**Abbreviations**: FIGO, International Federation of Gynecology and Obstetrics; LN, lymph node

### Relevance of ST14 and TMEFF1 expression in ovarian cancer

Among all 91 cases of ovarian cancer, 49 total cases showed high expression of ST14 and TMEFF1 at the same time, whereas 15 cases showed low expression at the same time (Table 8). Linear regression and correlation analysis showed that the expression intensity of ST14 and TMEFF1 were linearly correlated (*r* = 0.460, *P* < 0.001).

**Table 8:** Relevance of ST14 and TMEFF1 expression in ovarian cancer

TMEFF1	ST14		Total
	Low expression	High expression	
Low expression	15	18	33
High expression	9	49	58
Total	24	67	91

Note: The Spearman correlation coefficient rs was 0.460

### Interaction and co-expression of ST14 and TMEFF1 in ovarian cancer, and ST14 regulates the expression of TMEFF1

**Fig. 7:**
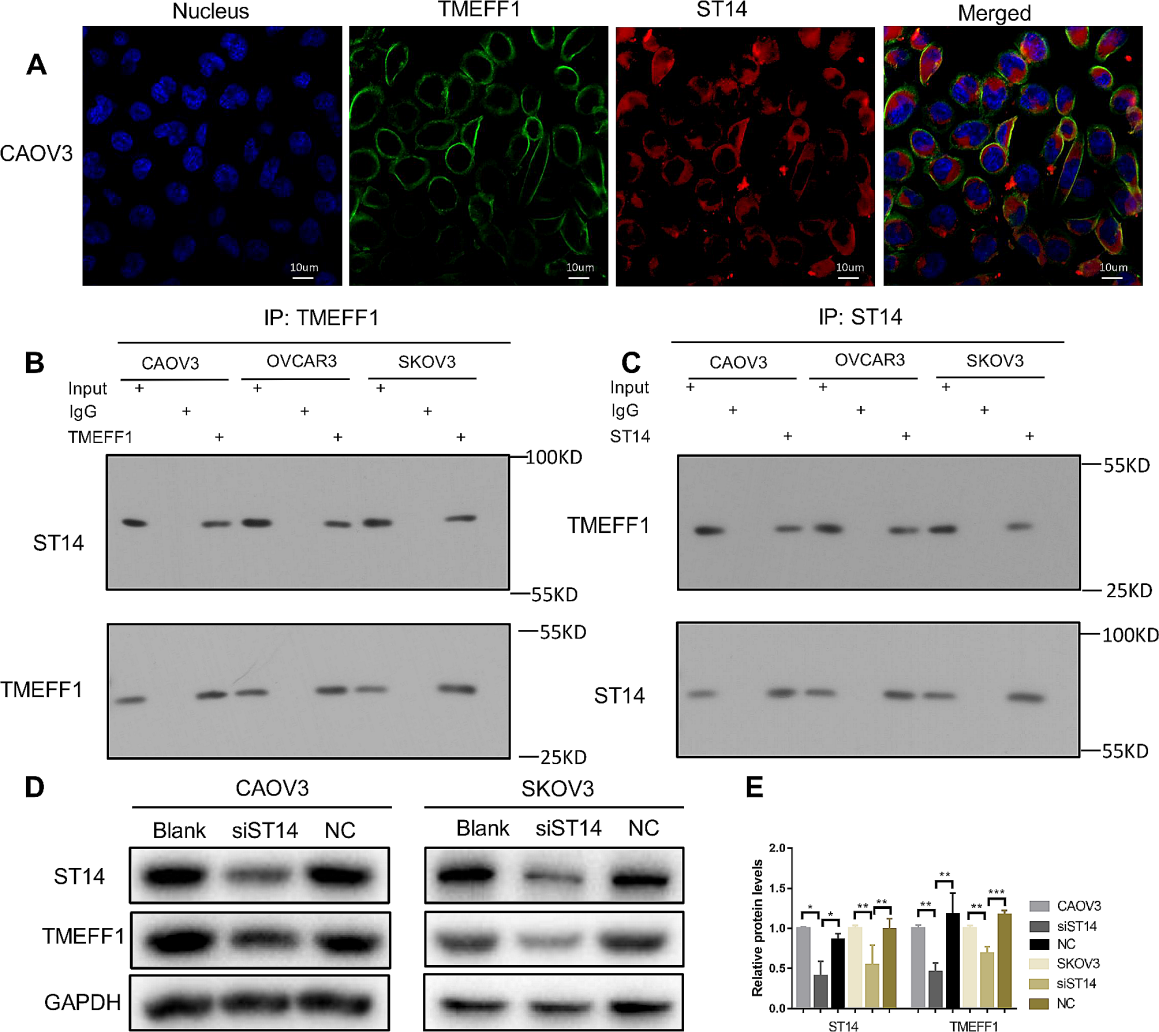
Co-localization and interaction of ST14 and TMEFF1 in ovarian cancer cells, and ST14 regulates the expression of TMEFF1. (**A**) Dual-labeled immunofluorescence technology was used to detect the co-localization of ST14 and TMEFF1 in ovarian cancer cell. Blue represents the nucleus, red represents ST14, green represents TMEFF1, and orange represents the co-localization of ST14 and TMEFF1 (original magnification, ×600). Pearson’s correlation coefficient (Rr) and Manders’ overlap coefficient (R) of the co-localization images are Rr:0.73, R:0.61. (B-C) The cell lysates of CAOV3, OVCAR3, and SKOV3 cells were immunoprecipitated with an anti-TMEFF1 antibody (**B**) and anti-ST14 antibody (**C**), and then, western blotting was performed with an anti-ST14 antibody and anti-TMEFF1 antibody. “Input” is the total cell lysate of CAOV3 cells. “IgG” is the negative control. (**D**) In the ovarian cancer cell lines CAOV3 and SKOV3, the expression of TMEFF1 decreased after knocking down the ST14 gene. (**E**) Quantification of ST14 and TMEFF1 normalized to GAPDH. Representative images and accompanying statistical plots are presented. Blank, blank control group, untreated original cells; siST14, ST14 gene knockdown group (through siRNA); NC, negative control group, negative gene (no sequence homology with ST14) knockdown group (through siRNA). Data are presented as the mean ± SEM (*n* = 3 per group). *P < 0.05, **P < 0.01, and ***P < 0.001

Through immunofluorescence double staining, we found that the co-localization of TMEFF1 and ST14 in the cell membrane of different ovarian tissues (Fig. 6B) and ovarian cancer cell line CAOV3 (Fig. 7A). The green fluorescently labeled TMEFF1 and the red fluorescently labeled ST14 overlapped based on orange fluorescence. Through immunoprecipitation, we found that ST14 and TMEFF1 interact in the ovarian cancer cells CAOV3, OVCAR3, and SKOV3 (Fig. 7B, C). Western blot results showed that knocking down ST14 in ovarian cancer CAOV3 and SKOV3 cell lines also decreased the expression of TMEFF1 (*P* < 0.05; Fig. 7D, E), indicating that ST14 regulates the expression of TMEFF1 in these cells.

### Interaction between ST14 and TMEFF1 promotes proliferation, invasion and migration of ovarian cancer

**Fig. 8:**
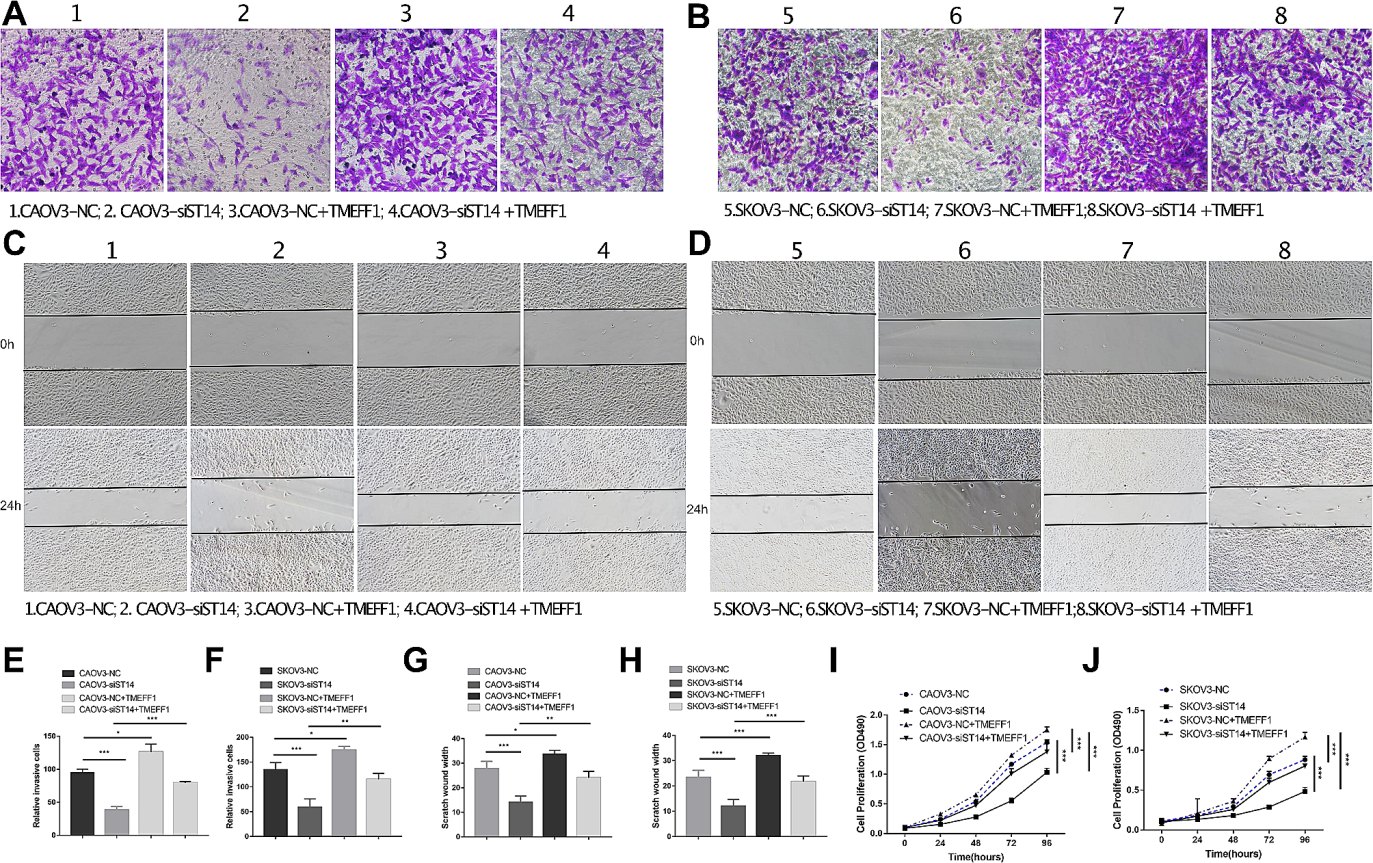
Interaction between ST14 and TMEFF1 promotes proliferation, invasion and migration of ovarian cancer. (**A**, **B**, **E**, **F**) The invasion capacities of ovarian cancer cells (CAOV3 and SKOV3) after downregulation of ST14 protein and addition of recombinant TMEFF1 active protein detected by Transwell assay. Number 1,2,3,4 respectively represents CAOV3-NC, CAOV3-siST14, CAOV3-NC + TMEFF1 and CAOV3-siST14 + TMEFF1; Number 5,6,7,8 respectively represents SKOV3-NC, SKOV3-siST14, SKOV3-NC + TMEFF1 and SKOV3-siST14 + TMEFF1. (**C**, **D**, **G**, **H**) The migration capacities of ovarian cancer cells (CAOV3 and SKOV3) after downregulation of ST14 protein and addition of recombinant TMEFF1 active protein detected by Wound healing assay. Number 1,2,3,4 respectively represents CAOV3-NC, CAOV3-siST14, CAOV3-NC + TMEFF1 and CAOV3-siST14 + TMEFF1; Number 5,6,7,8 respectively represents SKOV3-NC, SKOV3-siST14, SKOV3-NC + TMEFF1 and SKOV3-siST14 + TMEFF1. (**I**-**J**) The proliferation capacities of ovarian cancer cells (CAOV3 and SKOV3) after downregulation of ST14 protein and addition of recombinant TMEFF1 active protein detected by MTT assay. Data are presented as the mean ± SEM (*n* = 3 per group). *P < 0.05, **P < 0.01, and ***P < 0.001

In our preceding study, we investigated the impact of manipulating TMEFF1 expression on the biological behavior of ovarian cancer cells. We revealed that TMEFF1 overexpression facilitated ovarian cancer cell proliferation, migration, and invasion [10]. Building upon this foundation, we uncovered a significant interaction between ST14 and TMEFF1. In order to further detect the role of ST14 and TMEFF1 interaction in ovarian cancer cells, MTT, Transwell and Wound healing assays were performed. The results showed that the proliferation, invasion and migration abilities both decreased after downregulation of ST14 protein in CAOV3 and SKOV3 cells, and recovered after the addition of human recombinant TMEFF1 active protein by Transwell (Fig. 8A, B, E, F), Wound healing assays (Fig. 8C, D, G, H), and MTT (Fig. 8I-J). These results indicate that ST14 may affect the proliferation, invasion and migration of ovarian cancer cells by regulating the expression of TMEFF1.

## Discussion

Ovarian cancer is a gynecological malignant tumor associated with a poor prognosis and high morbidity and mortality [38]. Finding effective molecular indicators for early diagnosis and curative effect evaluations of ovarian cancer is very important.

ST14 was first detected in the culture medium of breast cancer cells cultured in vitro in 1993 [39]. Subsequently, it was found to play a role in other tumors. ST14 overexpression significantly enhances the invasion ability of colorectal cancer cells and affects the adhesion of cells to the extracellular matrix (ECM) [40]. ST14 activation can increase the migration and invasion of prostate cancer cells and promote tumorigenicity and tumor metastasis [41]. ST14 was also found to be a tumor suppressor gene. ST14 encoded protein can strengthen the intestinal epithelial barrier by promoting the formation of tight junctions. The ablation of ST14 in the epithelium of the small intestine of mice will lead to the rapid formation of colon adenocarcinoma [42]. However, in the study on ovarian cancer, the expression of ST14 resulted in different conclusions. Jin [43] found that compared to that in the normal ovarian epithelium, ST14 is highly expressed in ovarian cancer. Among subtypes, the expression of ST14 in serous cystadenocarcinoma is related to TNM stage and FIGO stage, and a later stage is linked to stronger expression. Tanimoto [44] and Oberst [45] found that compared with early ovarian cancer, ST14 expression is weaker in clinical specimens of advanced ovarian cancer, and ST14-positive patients showed a longer survival time. Therefore, the the expression and prognostic effect of ST14 in ovarian cancer is still controversial.

In this study, the results of Oncomine, UALCAN, and GEPIA database analysis showed that ST14 was significantly highly expressed in ovarian cancer and was related to the stage subgroup. We further validated this using ovarian cancer specimens and immunohistochemistry and found that ST14 is highly expressed in ovarian cancer, specifically in advanced stages and poorly differentiation groups, and is an independent risk factor for prognosis. Our results are consistent with Jin’s results. Studies have found that ST14 single nucleotide polymorphisms can independently predict a poor survival rate for breast cancer; that is, ST14 gene mutations affect the prognosis of tumors [46]. Therefore, we analyzed the relationship between such gene mutations and prognosis in ovarian cancer through the cBioPortal database, but found that ST14 is rarely mutated in ovarian cancer, probably due to less data, there is no significant difference, indicating that ST14 does not affect the progression of ovarian cancer through gene mutations.

The TMEFF1 gene was originally discovered as a gene encoding the secretory protein of the pituitary gland of Xenopus laevis. Subsequently, TMEFF1 was identified as a member of the CTA family [4]. The CTA family is involved in the occurrence and development of cancers and is currently a research topic of interest in cancer immunodiagnosis and immunotherapy [47, 48]. At present, the research of TMEFF1 in tumors is still limited. Initially, TMEFF1 was identified as a tumor suppressor gene in brain tumors [6]. High TMEFF1 expression has been detected in melanoma, liver cancer, and kidney cancer cell lines [9], but there have been no functional studies. We confirmed that TMEFF1 is an oncogene in ovarian cancer and endometrial carcinoma [10, 49]. TMEFF1 promotes cell proliferation, migration and invasion, inhibits apoptosis through MAPK and PI3K/AKT signaling pathways [10], and interacts with the tumor marker protein AHNAK in ovarian cancer [20]. We first discovered the interaction of TMEFF1 with ST14 in ovarian cancer. Two protein interactions have been found in autosomal recessive ichthyosis with hypoproliferation [9, 19]. The relationship between ST14 and TMEFF1 in tumors is still unknown. In this study, through immunohistochemistry, immunoprecipitation and double-labeled immunofluorescence assays we confirmed ST14 and TMEFF1 were expressed positively correlated, co-precipitated and co-localized in ovarian cancer.

ST14 was found to have similar biological functions to those of TMEFF1. GO analysis of ST14 and its related differentially expressed genes were involved in epithelial formation, cell adhesion, protein localization. ST14 is involved in the processes of cell adhesion and epithelial–mesenchymal transition (EMT), affects the adhesion of early colorectal cancer cells to the ECM and enhances invasion ability [40]. ErbB-2 signal transduction upregulates the activity of ST14, which in turn promotes the invasion of prostate cancer cells [50]. ST14 promotes the disintegration of cell connections and the formation of actin stress fibers, downregulates N-cadherin and α-SMA, enhances migration ability, and then causes epithelial cell EMT [51]. TMEFF1 is one of the core genes that regulate the EMT process [52]. By comparing the gene expression profiles of 14 paired ovarian serous adenocarcinoma samples with primary and metastatic (omental) samples, TMEFF1 was determined to be upregulated as an EMT indicator in the metastatic group [53]. During upregulation of the transcription factors Snail, Slug, and E47, which promote EMT in tumors, TMEFF1 is significantly upregulated [54]. These transcription factors are significant inducers of EMT and can strongly inhibit the expression of E-cadherin [54, 55]. In previous studies, we also found that TMEFF1 promotes the expression of N-cadherin, Vimentin, MMP2, and MMP9 in ovarian cancer cells, inhibits the expression of E-cadherin, and participates in the EMT process [10]. Based on the similar biological functions of TMEFF1 and ST14, we speculate that the interaction between them might affect their function in ovarian cancer.

We found that ST14 promotes migration and invasion of ovarian cancer cells by wound healing assay and Transwell assay in ovarian cancer. Our study showed that the proliferative, invasive and migratory abilities of ovarian cancer cells were inhibited after knockdown of ST14 protein, and those functions were restored by overexpression of TMEFF1 protein, suggesting that ST14 and TMEFF1 interact to form a protein complex, and ST14 can promote the proliferation, invasion and migration of ovarian cancer by regulating TMEFF1.

KEGG enrichment analysis of ST14 and its related genes showed enriched terms of tight junction, CAMs, p53 signaling pathway, NF-kappa B signaling pathway, and other pathways. It is now found that TMEFF1 and ST14 are closely related in biological functions. Zoratti found that the serine protease ST14 specifically cleaves the inactive pro-form of the hepatocyte growth factor (pro-HGF), promotes the release of HGF, binds c-Met, and then promotes the proliferation and invasion of inflammatory breast cancer cells [56]. As a transmembrane protein, TMEFF1 has an extracellular EGF-like domain, and the extracellular domain can be released as a soluble protein and activate erbB-4 tyrosine phosphorylation [57]. We speculated that ST14 may cleave and release extracellular EGF domain by binding to TMEFF1, then activate downstream receptor pathways. EGFR can mediate the activation of MAPK signaling pathway and AKT signaling pathway [58, 59]. Interestingly, both ST14 and TMEFF1 have been found to activate these pathways. In human epidermal tumors, ST14 can induce activation of the PI3K-Akt signaling pathway, and it can also cooperate with Ras-dependent signaling and independent signaling pathways to drive cancer [60]. We found that TMEFF1 promotes activation of PI3K/AKT and MAPK pathways in ovarian cancer to promote the malignant biological behavior of ovarian cancer cells [10]. Arano found that the membrane localization of TMEFF1 is crucial for its effect on cell migration, so the function of TMEFF1 may require interaction with ST14 on the membrane for activation [61]. In addition, both ST14 and TMEFF1 are involved in the TGF-β pathway, and TGF-β can promote the expression of both of them.

TGF-β upregulates the expression of ST14 through Smad2/Smad4-dependent transcriptional activation and promotes the EMT process [51]. TMEFF1 inhibits nodal signaling by competitively binding to Cripto-1 with ALK4, thereby mediating the functions of the TGF-β signaling pathway to regulate cell growth [62]. In the process of hair follicle regeneration, TMEFF1 is directly affected by Smad2/3, which is downstream of TGF-β signaling, to inhibit the activation of BMP signaling and relieve stem cell growth inhibition [52]. Therefore, we speculate that on the cell membrane, under the regulation of TGF-β signaling, ST14 may directly interact with TMEFF1, cleave and release the extracellular domain containing the EGF of TMEFF1, activate the downstream PI3K/AKT and MAPK pathways, and promote the invasion and metastasis of ovarian cancer cells. Thus, the specific mechanisms of interactions between ST14 and TMEFF1 affecting ovarian cancer still need to be studied in depth.

## Conclusion

In this study, we found that ST14 and TMEFF1 were overexpressed and interacted in ovarian cancer and both are independent risk factors for prognosis. ST14 can promote the proliferation,invasion and metastasis of ovarian cancer by regulating TMEFF1. Therefore, blocking the interaction site of ST14 and TMEFF1 protein may become a potential target for the treatment of ovarian cancer.

### Electronic supplementary material

Below is the link to the electronic supplementary material.

Supplementary Material 1

Supplementary Material 2

Supplementary Material 3

Supplementary Material 4

Supplementary Material 5

## Data Availability

All data are available in the manuscript.

## Abbreviations

CAMs: Cell adhesion molecules
CTA: Cancer testis antigen
DAPI: 4’,6-diamidino-2-phenylindole
DFS: Disease-free survival
DSS: Disease-specific survival
ECM: Extracellular matrix
EMT: Epithelial–mesenchymal transition
FIGO: Federation of Obstetrics and Gynecology
GO: Gene Ontology
GSEA: Gene set enrichment analysis
KEGG: Kyoto Encyclopedia of Genes and Genomes
OS: Overall survival
OV: Ovarian serous cystadenocarcinoma
PFS: Progression-free survival
SDS-PAGE: Sodium dodecyl sulfate-polyacrylamide gel electrophoresis
TCGA: The Cancer Genome Atlas
TTSPs: Type II transmembrane serine proteases
