# Supplementary material for: ST14 interacts with TMEFF1 and is a predictor of poor prognosis in ovarian cancer
Source: BMC Cancer. 2024 Mar 11;24:330. doi: 10.1186/s12885-024-11958-8 (PMC10929089; doi:10.1186/s12885-024-11958-8)
Supplement: Supplementary file 1 — Supplementary Material 1 [file 12885_2024_11958_MOESM1_ESM.docx]

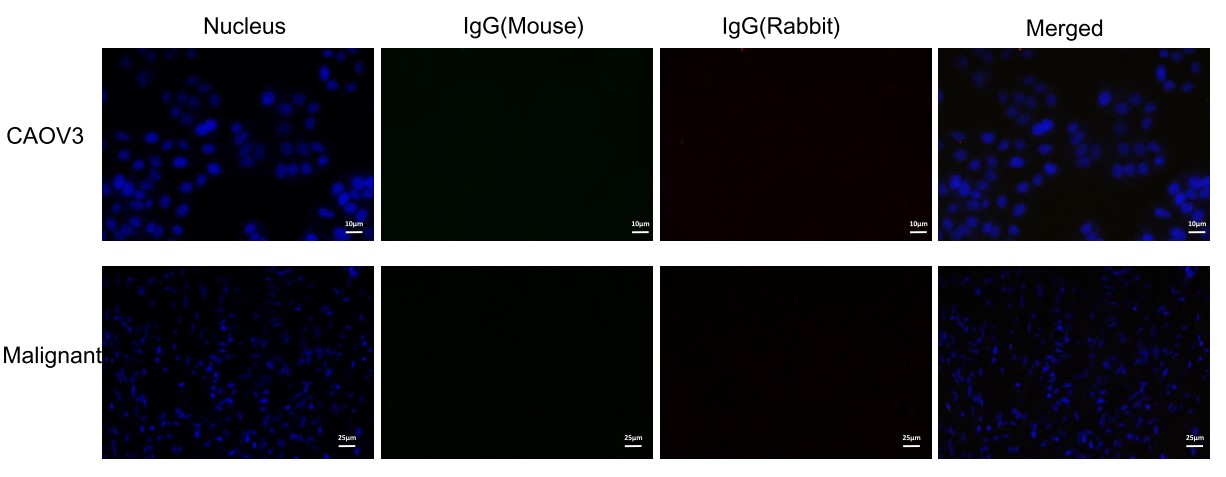
Figure S1. The negative control of dual-labeled immunofluorescence technology.

The primary antibody (TMEFF1 and ST14) was replaced with rabbit or mouse IgG as a negative control. Blue represents the nucleus, red represents rabbit, green represents mouse (original magnification, ×600, ×400).
