# Supplementary material for: ST14 interacts with TMEFF1 and is a predictor of poor prognosis in ovarian cancer
Source: BMC Cancer. 2024 Mar 11;24:330. doi: 10.1186/s12885-024-11958-8 (PMC10929089; doi:10.1186/s12885-024-11958-8)

Figure 1G
ST14

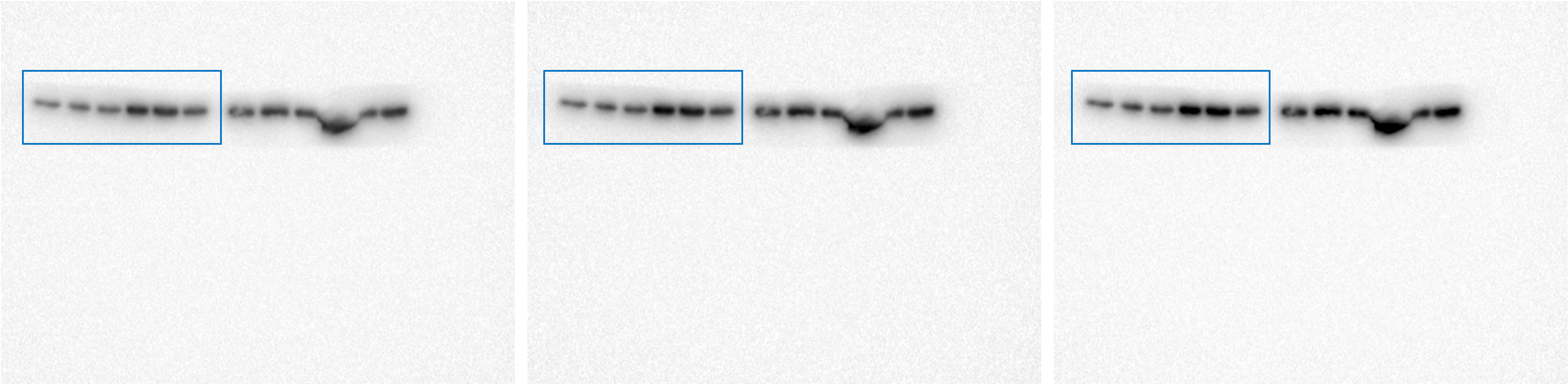

Figure 1G
TMEFF1

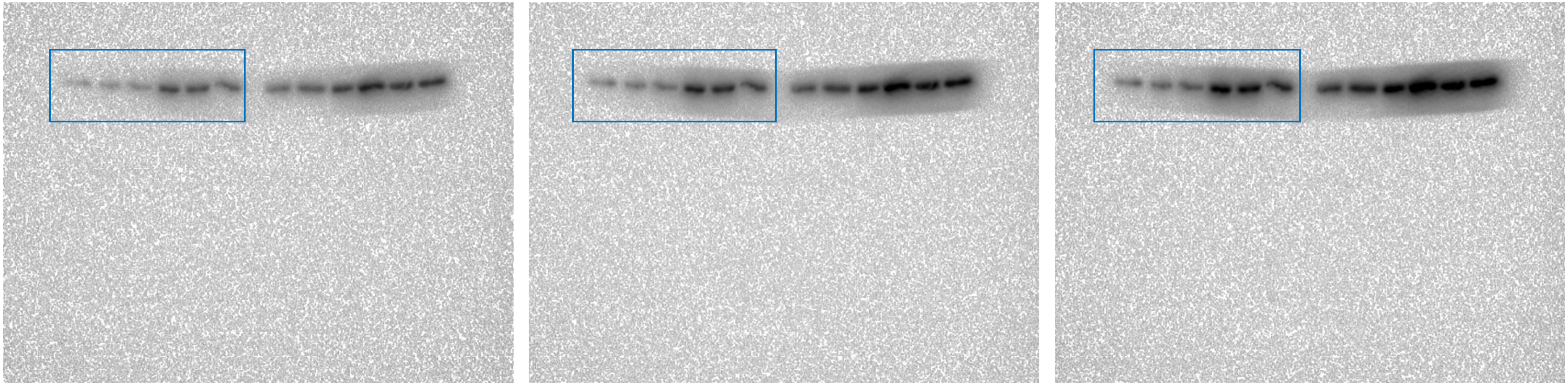

Figure 1G
GAPDH

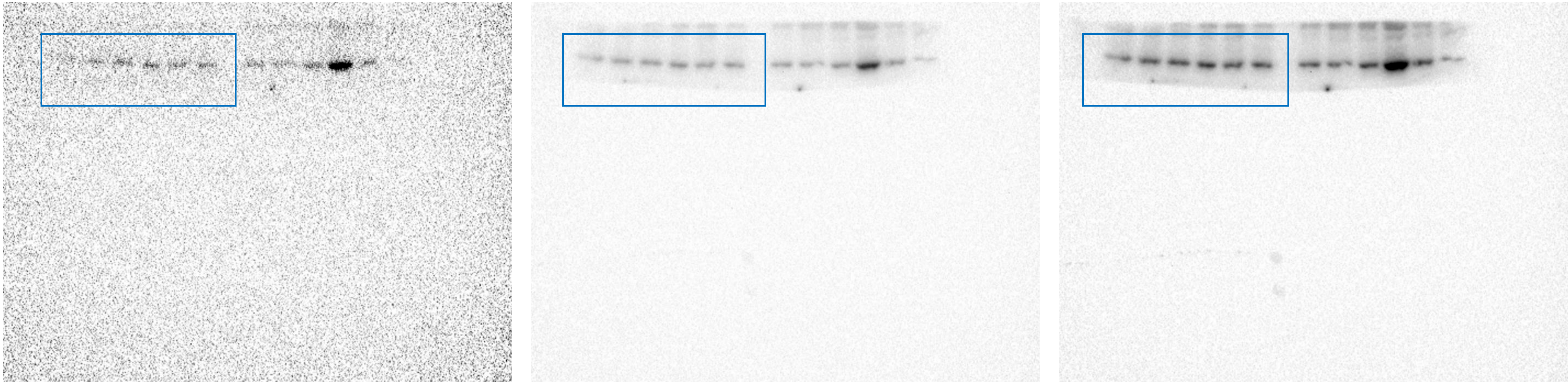

Figure 7B
IP-TMEFF1
ST14

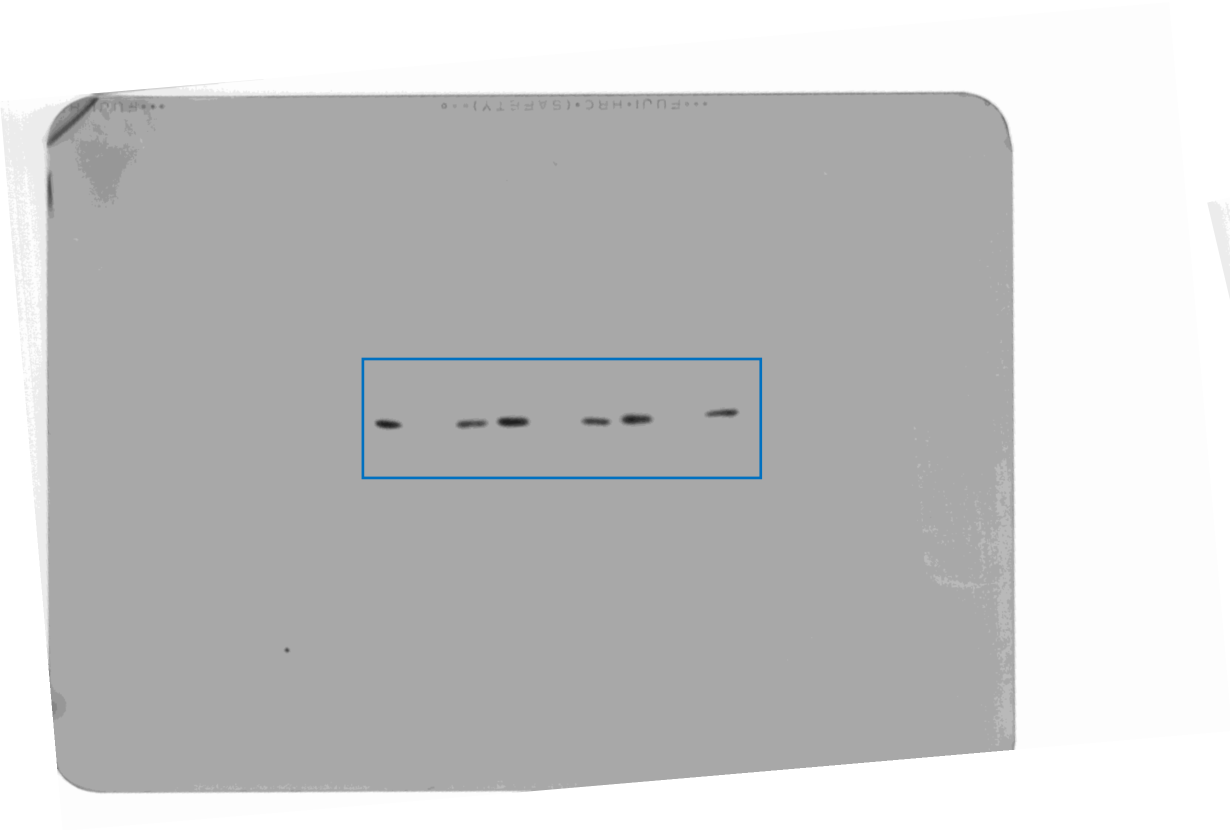

Figure 7B
IP-TMEFF1
TMEFF1

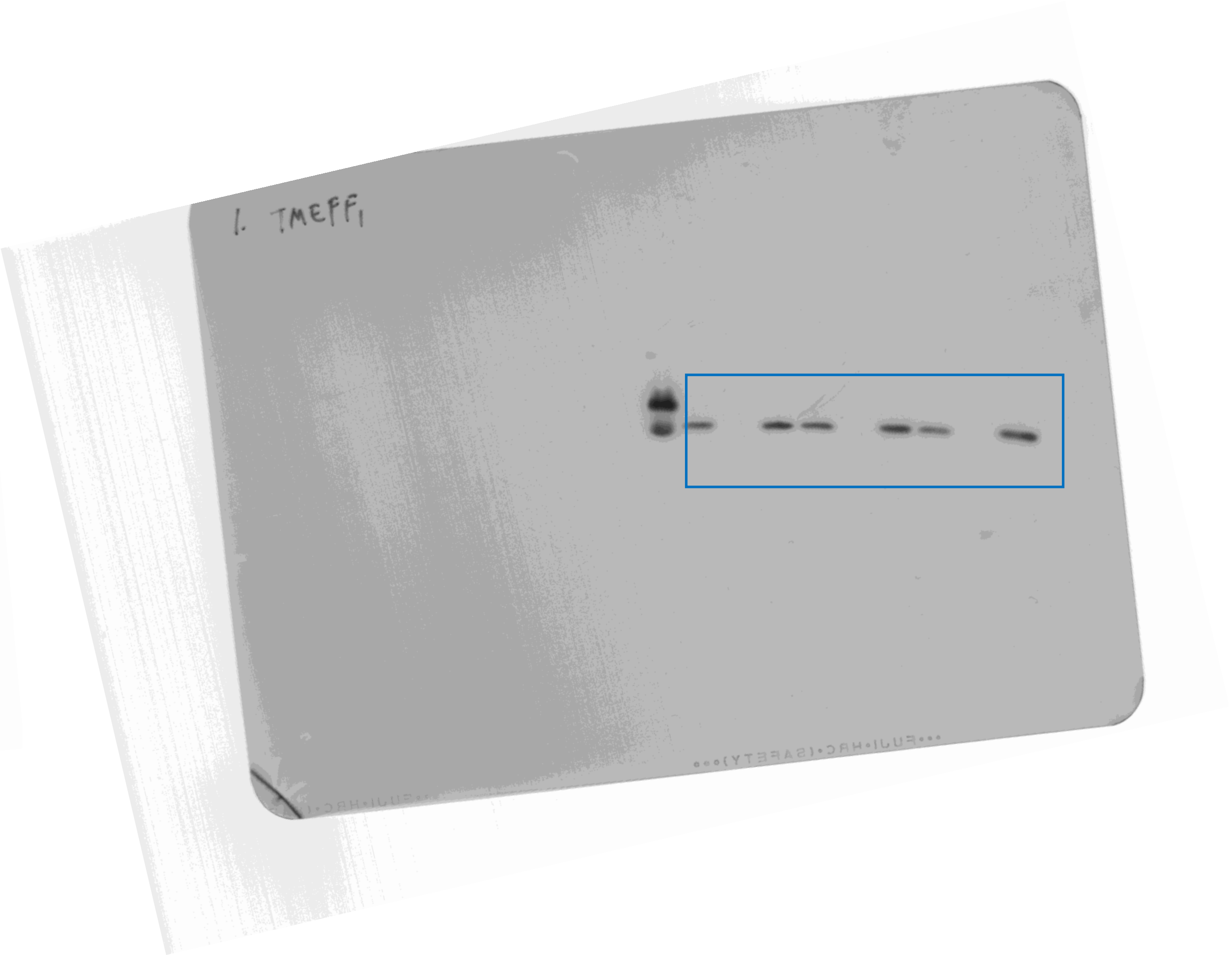

Figure 7C
IP-ST14
TMEFF1

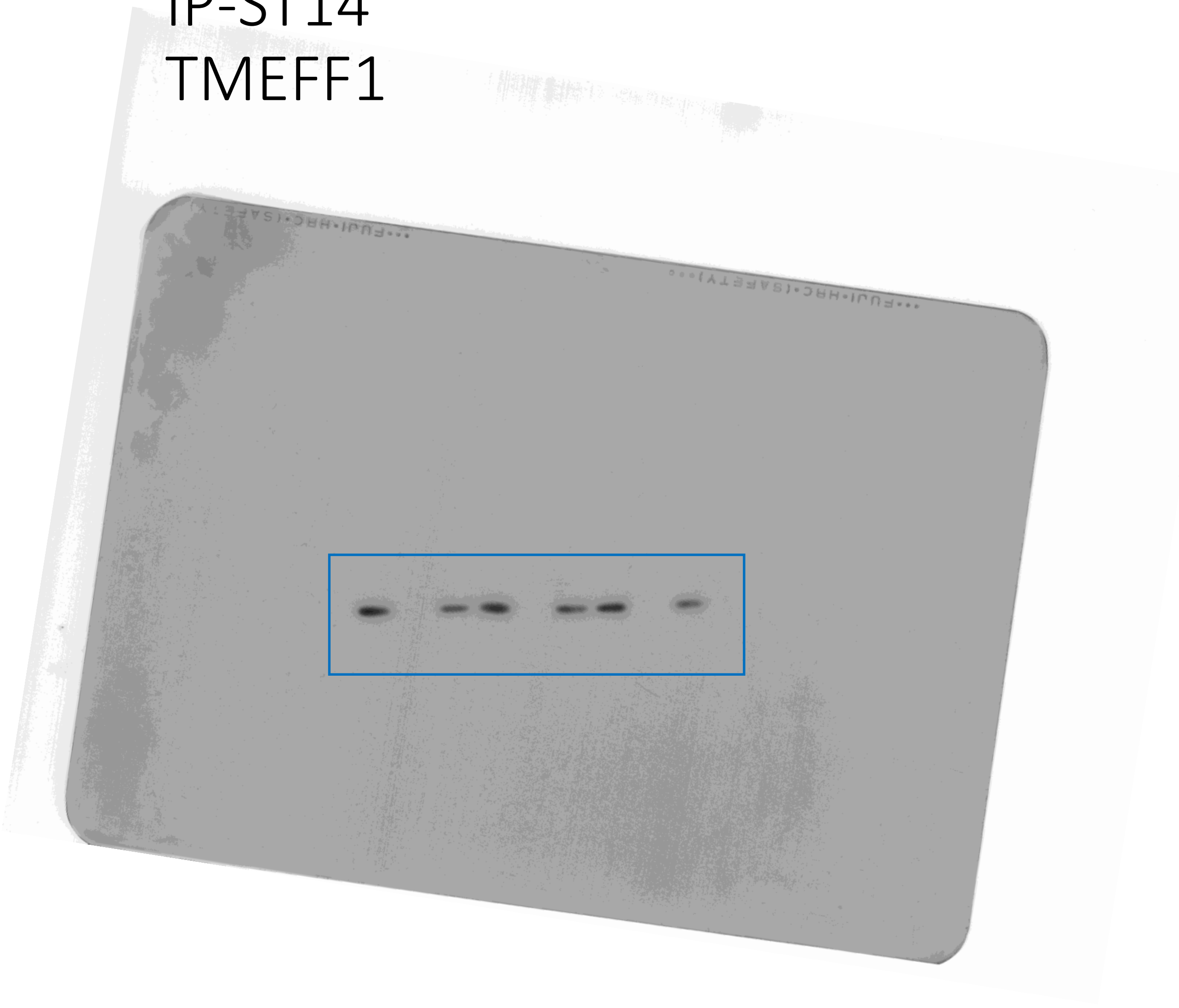

Figure 7C
IP-ST14
ST14

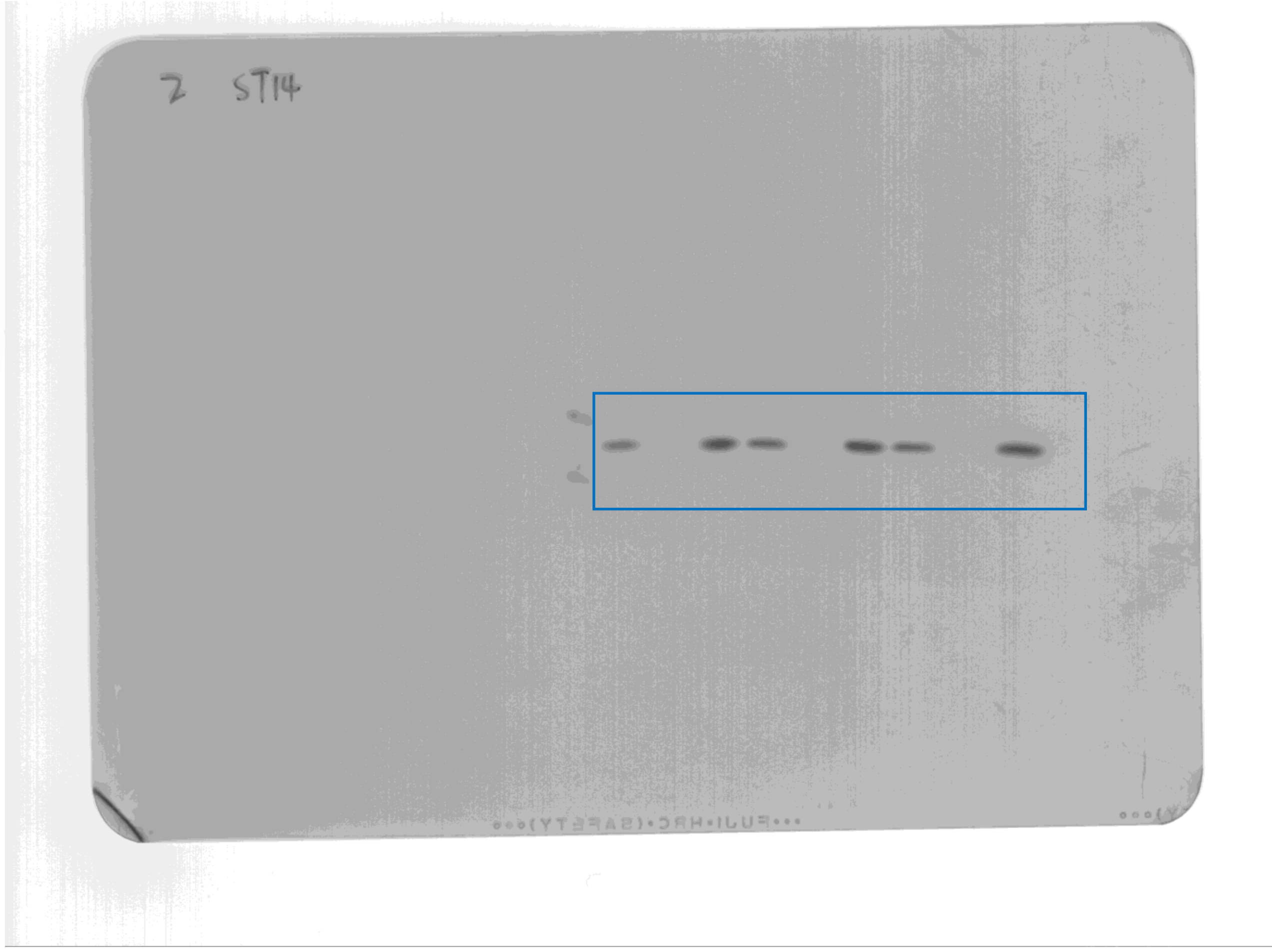

Figure 7D
CAOV3-ST14

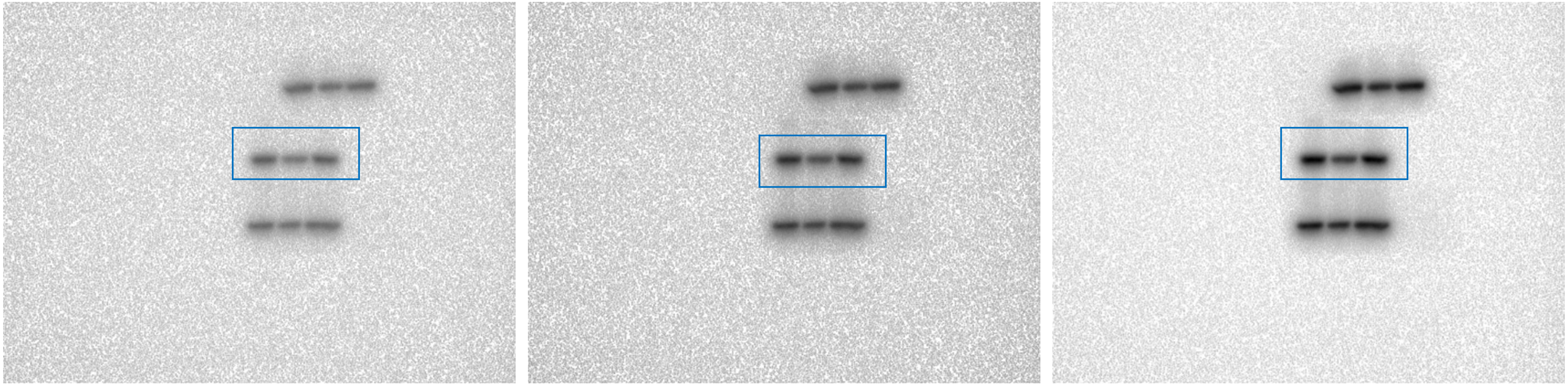

Figure 7D
CAOV3-TMEFF1

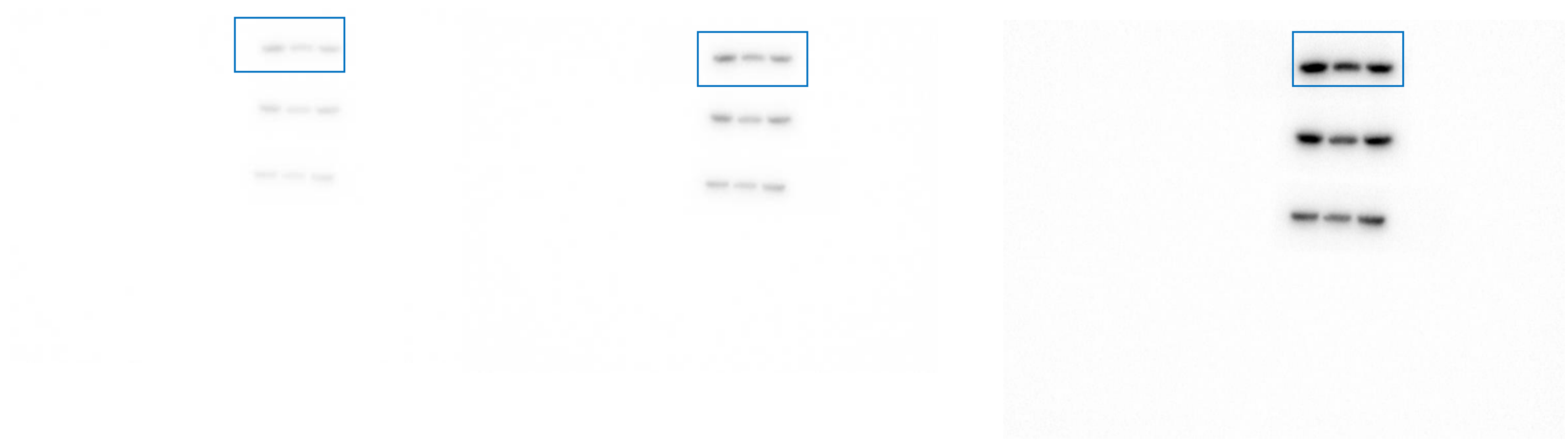

Figure 7D
CAOV3-GAPDH

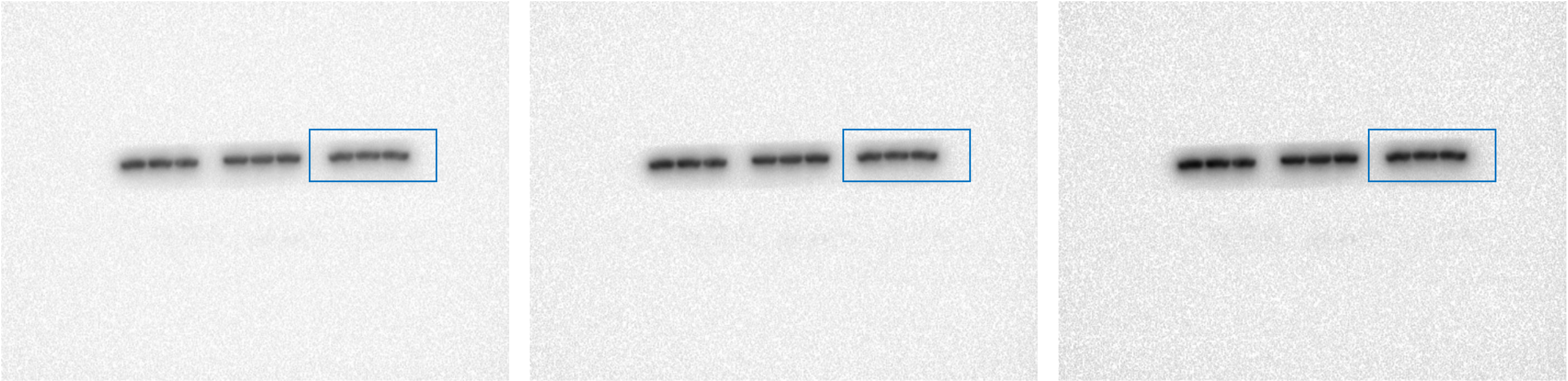

Figure 7D
SKOV3-ST14

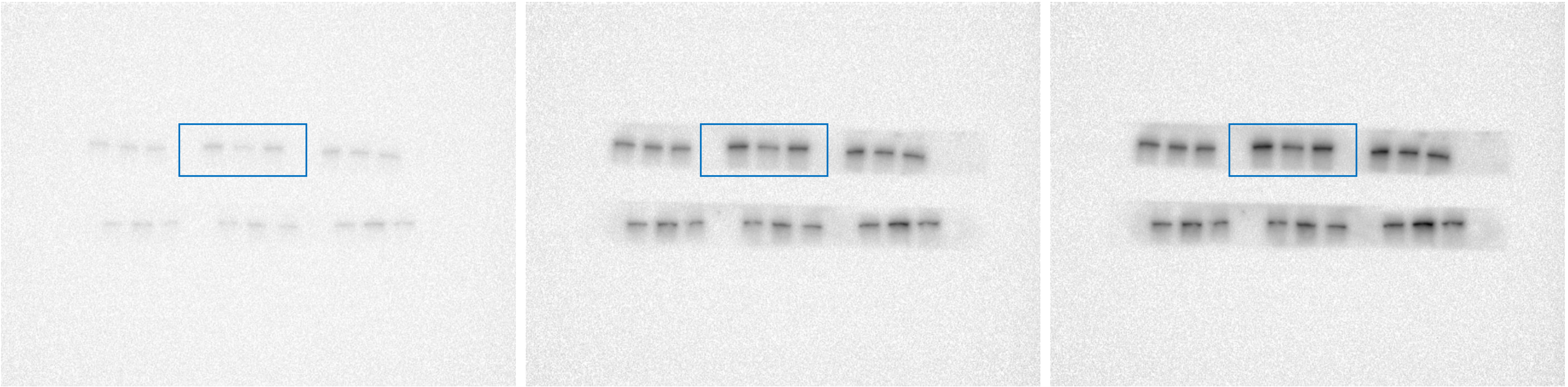

Figure 7D
SKOV3-TMEFF1

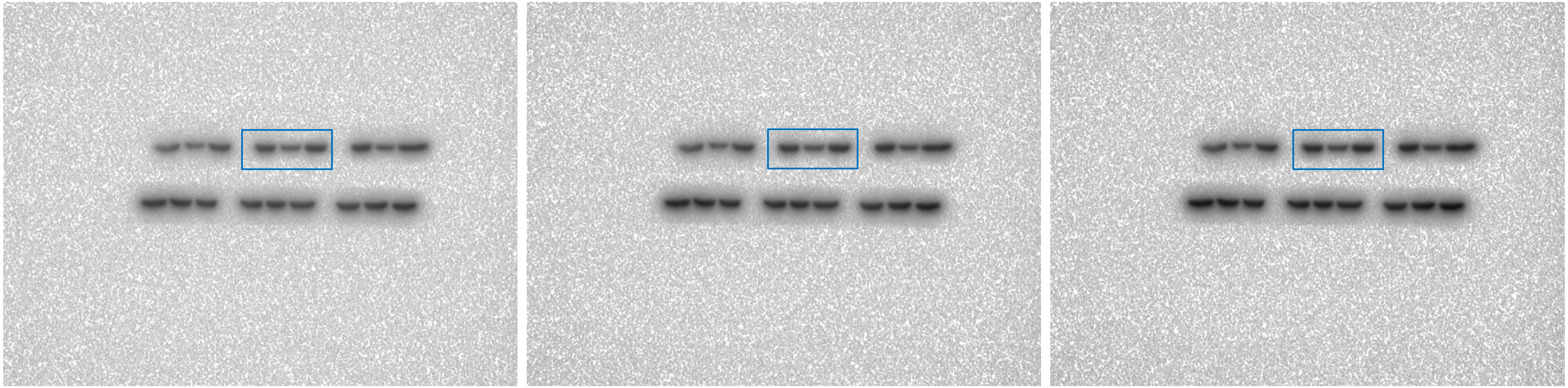

Figure 7D
SKOV3-GAPDH

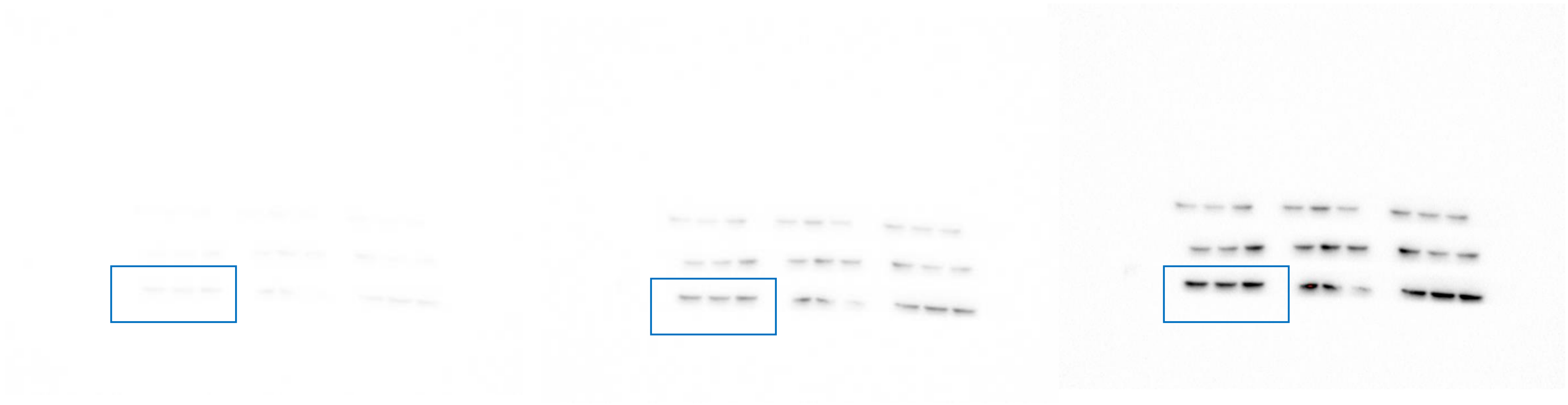

Supplement: Supplementary file 5 — Supplementary Material 5 [file 12885_2024_11958_MOESM5_ESM.pdf]
